# An integrated fuzzy multi-criteria decision making and mixed-integer linear programming optimization framework for project risk assessment and response strategy selection: A case study of a liquefied petroleum gas pipeline construction project

**DOI:** 10.1371/journal.pone.0357560

**Published:** 2026-09-24

**Authors:** Mohammad Senisel Bachari, Asgar Khademvatani, Mahdi Iranfar

**Affiliations:** Department of Energy Economics and Management, Tehran Faculty of Petroleum, Petroleum University of Technology, Tehran, Iran; Philadelphia University, JORDAN

## Abstract

Effective project risk management helps to reduce negative outcomes, especially in construction projects. Because project risk management involves many different parts, an integrated approach is needed to cover all phases. This study proposes an integrated framework for risk planning, identification, assessment, and response strategy selection, addressing major weaknesses in traditional risk management methods. The framework systematically identifies risks, their characteristics, and their impact zones. These elements help to evaluate each risk thoroughly. Risk assessment is performed using fuzzy multi-criteria decision-making, fuzzy SWARA (Step-wise Weight Assessment Ratio Analysis) and fuzzy WSM (Weighted Sum Model). The selection of risk response strategies is then formulated as a mixed integer linear programming (MILP) optimization model. This produces an optimal risk response strategy that reduces threats and takes advantage of opportunities. To provide a proof-of-concept illustration of the proposed integrated framework, we present a case study utilizing empirical data from a real‑world liquefied petroleum gas (LPG) pipeline construction project. The findings identify 20 distinct risks and 12 viable risk response strategies. The optimization component successfully selects six out of twelve response strategies. This achieves a 38.9% reduction in total risk value while meeting the project’s constraints.

## Introduction

Industries, businesses, and organizations undertake projects because they offer opportunities for value creation, value capture, and broader contributions to the projectivization of economies and societies. Effective project governance—encompassing portfolio management, project sponsorship, and project performance optimization—can generate significant organizational value (e.g., [1,2]). According to the Project Management Institute (PMI) —the issuing body of the PMBOK® Guide, a globally recognized standard for project management—projects are temporary endeavors aimed at delivering positive transformations, such as specific products or services [3]. Project management is the structured process through which these transformations are achieved, and project management offices play a crucial role in enhancing organizational performance [4]. Successful project execution improves organizational efficiency and productivity [5].

However, the successful project execution depends heavily on robust risk management practices. Projects are exposed to various risks at multiple stages, which can adversely affect quality, cost, scope, leading to delays and deviations from the project schedule (e.g., [6,7]). This is particularly construction and engineering projects [8]. The literature presents multiple definitions of risk [9–12]. A common but often overlooked characteristic of is dual nature of risks; they can represent either threats or opportunities for projects. Despite this duality, risks are predominantly associated with negative outcomes. This study considers both negative and positive risks [13]. Once a risk is triggered, it can impact time, cost, quality, scope, and overall objectives [14].

**Fig 1:**
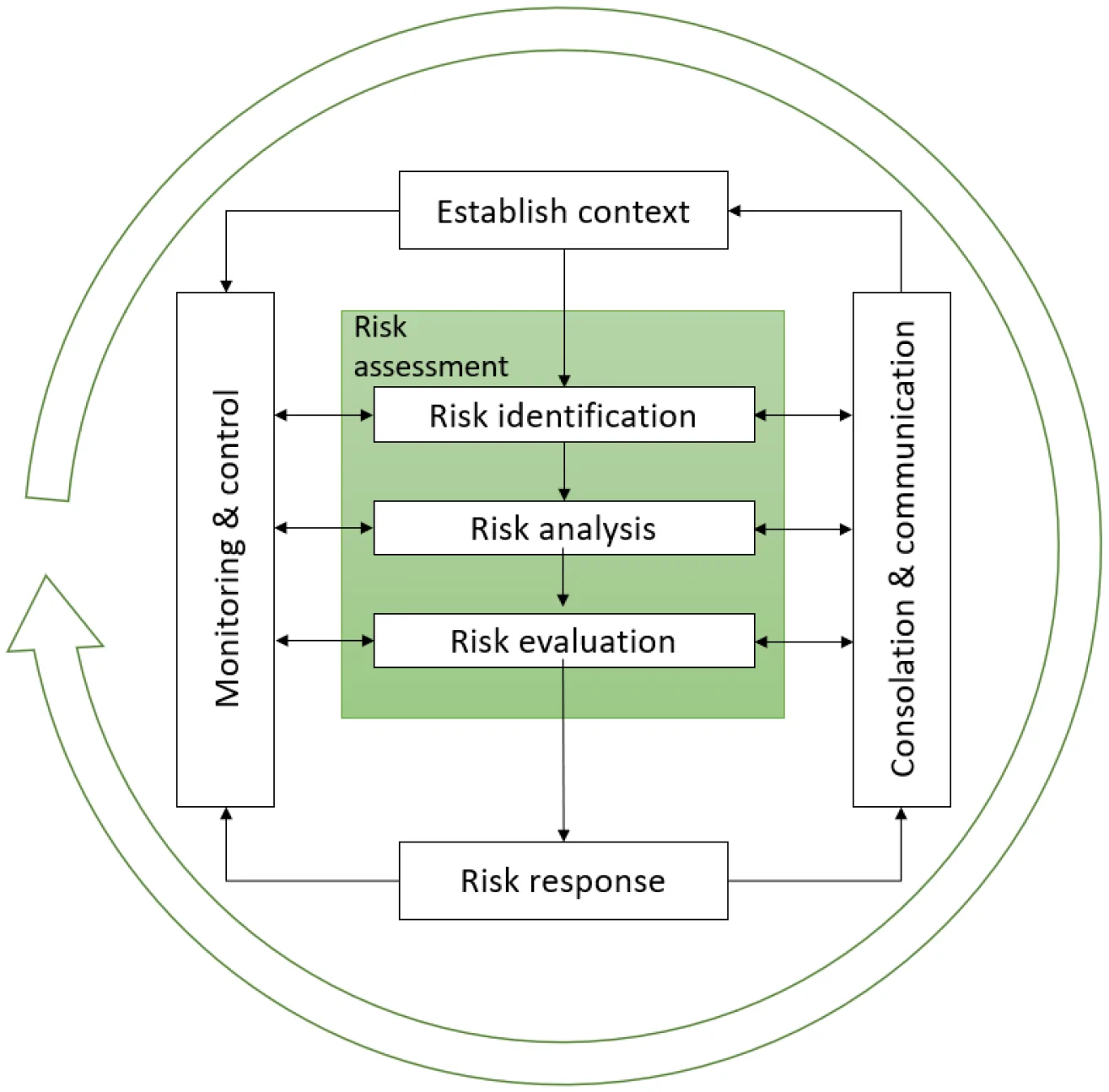
Risk management cycle [22].

Risks are classified based on diverse criteria, such as source, nature, originating entity, or hierarchical level (e.g., [15,16]). Traditional risk impact assessment relies on likelihood and severity. Severity is influenced by factors including human resources, workplace conditions, materials, and equipment, which can be challenging to quantify [17]. Although probability and severity are primary factors for risk scores, other characteristics—such as risk detection, manageability, persistency, predictability, ubiquity, and uniqueness—are frequently overlooked (e.g., [15,18]). Additionally, risks interdependencies, where one risk influences another’s probability or impact, can create cascading effects that are often neglected [19].Managing risks is crucial across various sectors. According to the PMI’s Guide to the Project Management Body of Knowledge (PMBOK® Guide) [3]) is typically a composition of multiple knowledge domains, and Project Risk Management (PRM) is one of the key knowledge domains in project management; PRM is typically includes Risk Planning, Identification, Analysis, Response, and Monitoring/Control [3,20]. In practice, uncertainties and risks are inherent to construction projects. PRM focuses on identifying and mitigating risks [21]. Similarly, the ISO 31000(2018) risk management standard [22] proposes a comparable structure (Fig 1), in which risk assessment—comprising identification, analysis, and evaluation—is continuously linked to risk response strategies and monitoring. Risk assessment (identification and analysis) and risk responses are critical components of both standards.

Risk identification traditionally relies on expert judgment using methods like brainstorming, interviews, checklists, risk breakdown structure (RBS), and the Delphi method (e.g.[23–27]). Risk analysis involves measuring, evaluating, and prioritizing risks through qualitative and quantitative methods (e.g., [28,29]). Academically, risk assessment studies have gained increasing attention. Ahmed et al. [30] reviewed techniques for project risk management and identified a trend toward more quantitative methods. Afzal et al. [31] examined artificial intelligence-based risk assessment methods, concluding that capturing risk interdependencies remains a challenge. Al Qudah et al. [32] conducted a bibliometric analysis of construction risk management literature, highlighting the growing use of MCDM and optimization techniques. Despite this progress, these studies do not propose an integrated framework that combines risk assessment with response selection, nor do they consider risk characteristics beyond probability and severity. The present study addresses these gaps.

**Fig 2:**
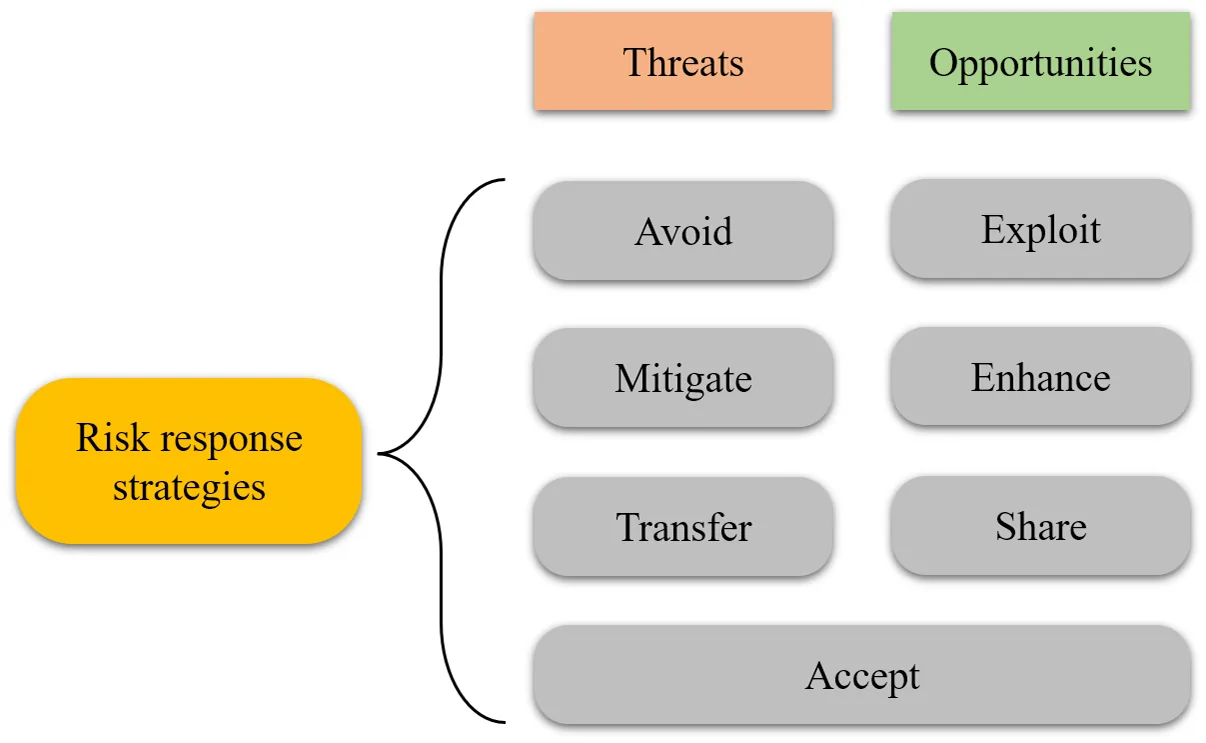
Risk response strategy categories [33].

Identifying and measuring risks constitutes only part of the risk management process; there remains a critical need to respond appropriately to these risks. According to the risk management standards [22], the next step after risk assessment is risk response selection. Risk response selection is the process of assigning resources to countermeasures aimed at reducing the impact of risks. Project response strategies are essential for proactively managing uncertainties that could affect project objectives.. The categorization of risk response strategies is illustrated in Fig 2.

Effective selection of risk response strategies helps to minimize project disruptions, reduce financial losses, and enhance decision-making (e.g., [34,35]). Risk response methodologies are generally categorized into four groups: (a) zonal-based approaches, (b) trade-off approaches, (c) work break structure (WBS)-based approaches, and (d) optimization approaches. The fourth group (optimization) is characterized by greater precision, and recent studies increasingly favor these methodologies (e.g., [36,37]). Optimization approaches are typically applied in isolation from risk assessment. This gap between risk assessment studies (Table 1) and response selection studies (Table 2) motivates the need for an integrated framework that links risk properties to response selection. In recent years, project risk management research has increasingly shifted toward the use of optimization-based and computational decision-support approaches to support risk mitigation planning under resource constraints. Recent advances have introduced a range of techniques beyond traditional scoring methods. Studies have explored mixed-integer programming, stochastic optimization, and hybrid multi-criteria decision-making (MCDM) frameworks to prioritize risks and select mitigation strategies. Specifically, MCDM approaches such as TOPSIS, AHP, and SWARA have been widely applied to rank and evaluate risks (e.g., [30–32]). Stochastic and simulation-based methods (e.g., Monte Carlo simulation, Bayesian networks) have been used to capture uncertainty in risk parameters (e.g., [19,36,37]). Mixed-integer linear programming (MILP) has emerged as a powerful tool for selecting risk response strategies under resource constraints (e.g., [37]). However, much of the existing literature either focuses on risk ranking without explicitly optimizing response portfolios, or formulates optimization models that lack structured integration of expert-based risk properties and project-stage considerations. Furthermore, most existing studies treat risk assessment and response selection as separate processes; few integrate both phases within a single framework, and even fewer incorporate risk characteristics beyond probability and severity, the dual nature of risks (threats and opportunities), or interdependencies among risks. The framework proposed in this paper addresses these gaps by integrating fuzzy MCDM for risk assessment with MILP for response selection, while explicitly modeling risk interdependencies and dual risk effects. Despite the recognized importance of project risk management, several critical gaps remain unaddressed in existing research and practice. First, although risks have a dual nature (threats and opportunities), most frameworks focus predominantly on negative risks. Second, risk assessment traditionally relies only on probability and severity, ignoring other influential characteristics such as detectability, persistency, and interdependencies. Third, while optimization approaches for risk response selection are gaining traction, no integrated systematic framework yet exists that combines comprehensive risk assessment (considering multiple risk characteristics and interdependencies) with the simultaneous selection of risk response strategies under project constraints. Fourth, existing methods often treat risk assessment and response selection as separate sequential processes rather than as an integrated system. These gaps lead to suboptimal risk management, particularly in complex projects like construction and engineering, where overlooked interdependencies and positive risks can significantly affect project outcomes. In particular, relatively few studies provide a transparent linkage between risk assessment outputs and a formal optimization model for selecting an optimal combination of mitigation strategies. While these approaches select response strategies based on risk scores, an integrated systematic risk identification, analysis and response that accounts for risk values derived from risk characteristics has not yet been established. Although there are studies which present methodologies for risk strategy selection, they do not incorporate a risk assessment approach and often overlook important characteristic of risks (e.g., risk interdependencies, dual risk effect, etc.).

**Table 1:** A summary of relevant risk assessment studies.

Author/ year	Main theme	Method	Project context
**[47]**	This study explores the applica33tion of robustness as a method for managing risks in developing complex petroleum fields.	Multi-Attribute Utility Theory (MAUT)	Energy sector
**[48]**	This study refines the risk–risk trade-off method to assess preferences for mitigating mortality and morbidity risks.	pre-survey learning experiments and risk-framing techniques	Construction
**[49]**	This paper proposes a fuzzy model incorporating risk-based TOPSIS for preventive maintenance planning	Fuzzy reliability model and risk-based TOPSIS methodology	Manufactory
**[50]**	This article develops an integrated decision support methodology for analyzing risk factors in oil and gas construction projects	artificial neural network (ANN) for risk effect prediction	Energy sector
**[14]**	This research identifies and models risk factors in construction projects, particularly emphasizing the impact of internal and external risks on project success.	Statistical analysis using Partial Least Squares Structural Eq. Modeling	Construction
**[51]**	This paper simulates a groundbreaking model the evolution of project portfolio risk by incorporating the dynamics of risk interactions and the contagious nature of risks.	model validated through computational simulations.	Organizational
**[52]**	This study proposes a novel scenario reduction method for optimization problems that utilizes higher moment coherent risk measures.	Scenario reduction technique	Organizational

Source: Research findings.

**Table 2:** A summary of relevant studies for risk response strategies.

Author/ year	Main theme	Method	Project context
**[37]**	This study proposes an innovative optimization model for selecting effective risk response strategies.	Optimization model	–
**[60]**	This research develops a systematic approach for generating effective project risk response strategies.	Case-based reasoning	Construction
**[19]**	This article introduces an optimization model for selecting risk response strategies that takes into account the interdependencies among risks.	Optimization model	–
**[61]**	This paper develops a mathematical model to optimize the selection of risk response strategies specifically for construction projects.	Mathematical programming	Construction
**[59]**	This work proposes a risk response selection model that integrates the correlations among interdependent risk factors.	Grey K-shell algorithm	–
**[33]**	This study presents a mathematical optimization method for selecting both local and global risk responses across the entire portfolio.	Mathematical optimization	Organizational
**[62]**	This research introduces a method for identifying and selecting optimal risk response strategies specifically tailored for design projects.	FMEA and Matrix of Alliances and Conflicts	IT & software
**[36]**	The study develops a comprehensive portfolio-wide approach to optimize the selection of response strategies for effectively managing risks associated with external stakeholders.	Bayesian influence diagram combined with multi-objective optimization.	Organizational.
**[63]**	This research introduces a comprehensive framework and optimization model for selecting risk response strategies, highlighting the interdependence of various risks.	Optimization model with a maximization utility function	Disaster management
**[64]**	This paper presents a simulation–optimization model to dynamically select risk response strategies.	System Dynamics and optimization-based simulation model	Construction

Source: Research findings.

**Fig 3:**
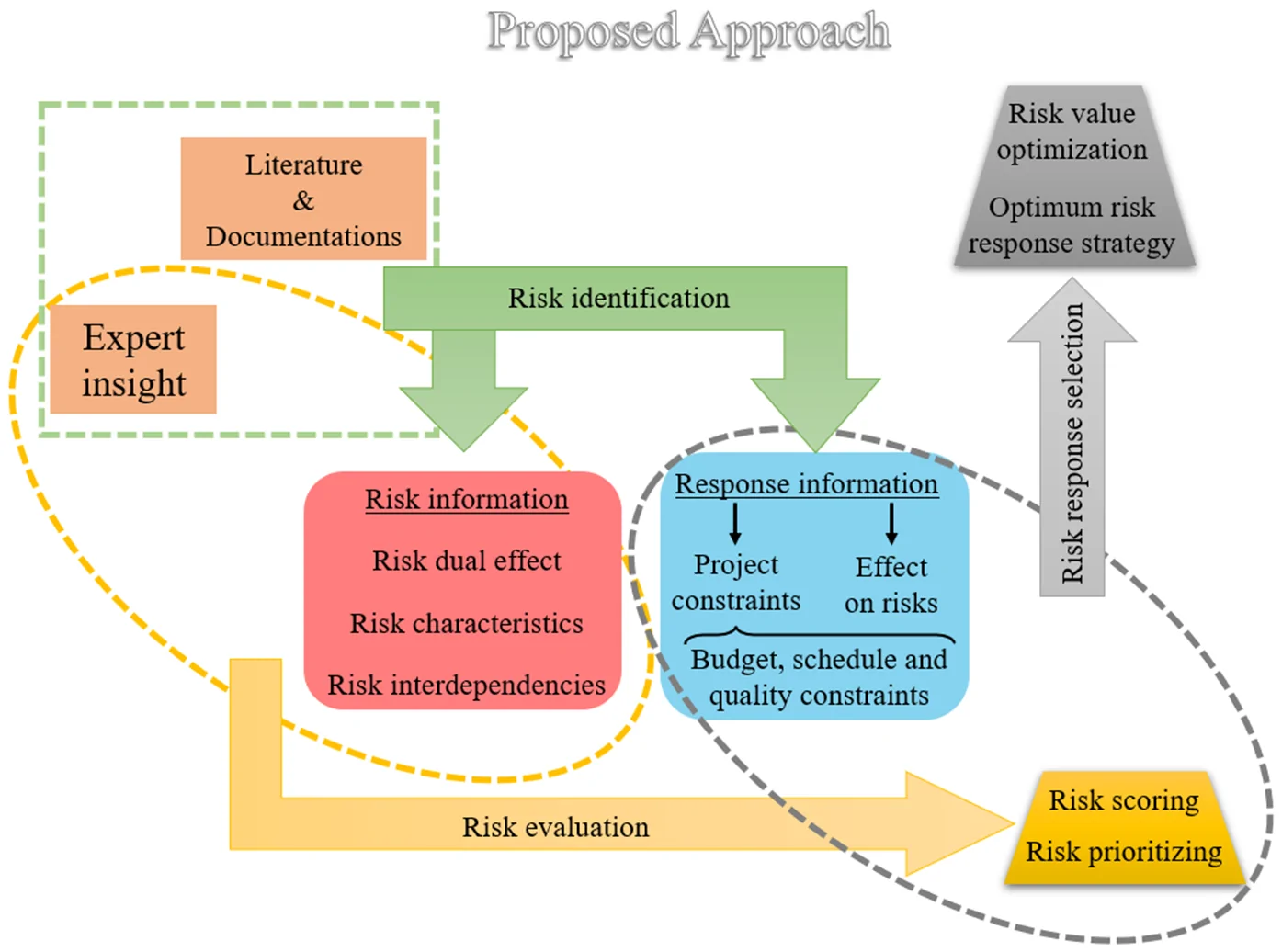
Structure of proposed research approach, distinguishing risk properties from risk management process phases. Source: Authors’ findings.

The literature gap identified above — specifically, the lack of integration between risk assessment and response selection — directly informs the design of our proposed framework. Fig 3 translates this gap into a conceptual structure by mapping risk properties onto risk management process phases. The choice of risk variables, dual effect, characteristics, and interdependencies reflects the overlooked risk attributes identified in the literature review (see Table 1). The choice of risk identification, evaluation, and response selection reflects the key phases of the risk management cycle where integration is currently lacking (see Table 2). Thus, Fig 3 is not arbitrary; it operationalizes the specific gaps identified in the literature. Given the importance of effective project risk management, it is essential to adopt an integrated systematic approach to manage these critical processes. The goal of this study is to propose such an approach. Fig 3 provides a high-level overview of the proposed research approach.

It is important to distinguish between two conceptual dimensions: (1) risk information(properties) — including risk variables, dual effect, characteristics, and interdependencies—which describe the intrinsic attributes of risks; and (2) risk management process phases — including risk identification, evaluation, and response selection — which represent the sequential steps of the risk management cycle. Fig 3 aligns these two dimensions in a schematic format to illustrate how risk properties inform each risk management process phase.

**Fig 4:**
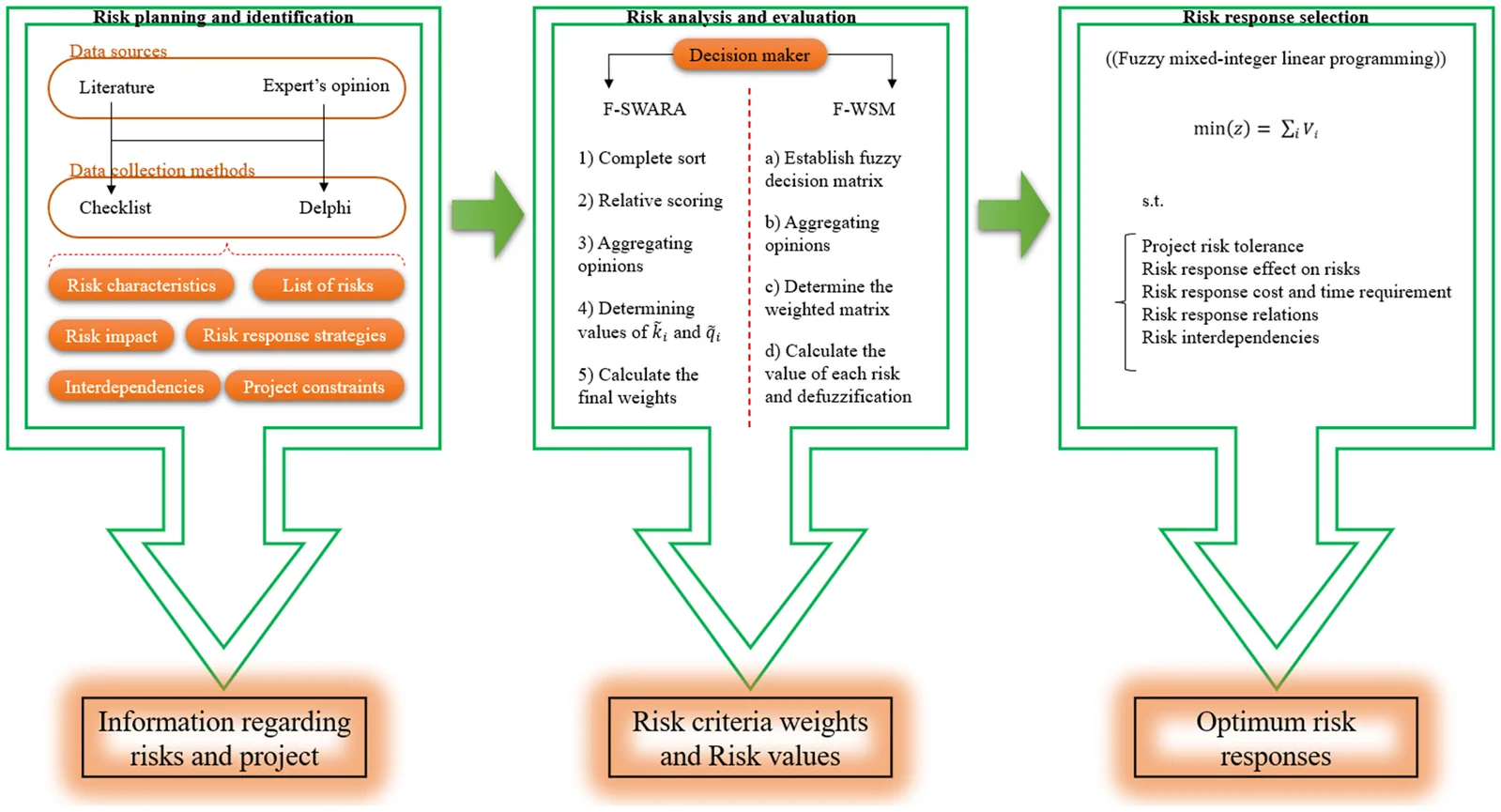
A proposed integrated optimization framework — a structured analytical workflow. The framework is implemented programmatically using Python with the CPLEX library (via Docplex) for the optimization stage (Stage 3). Stage 2 (F-SWARA and F-WSM) is automated via Python scripts that handle fuzzy aggregation, weighting, and defuzzification. While the figure depicts logical steps for clarity, the actual execution is fully computational. Source: Research findings.

The conceptual matrix in Fig 3 is operationalized into a step-by-step methodological workflow in Fig 4, which translates the abstract dimensions (risk properties and process phases) into concrete analytical stages: risk planning and identification (Stage 1), risk assessment using fuzzy MCDM (Stage 2), and response strategy selection using mixed-integer linear programming (Stage 3).In this framework, risk properties are systematically mapped to each phase of the risk management process to ensure that risk characteristics inform decision-making at every stage. Therefore, this study aims to propose an integrated systematic approach for identifying, evaluating, and prioritizing risks, and for selecting appropriate risk response strategies that optimize risk value while respecting project constraints. The study systematically evaluates and prioritizes risks through a structured multi-stage process: (1) risk criteria are weighted using the Fuzzy Step-wise Weight Assessment Ratio Analysis (F-SWARA) method (see Section 3, Eqs. (1)– (5) and Tables 3,4); (2) each risk is assessed against six criteria (probability, detectability, manageability, cost, time, quality) using the Fuzzy Weighted Sum Model (F-WSM) (see Section 3, Eqs. (6)– (10) and Table 5); and (3) risks are ranked based on their defuzzified scores (Eq. (5)). This systematic evaluation, which extends beyond conventional probability-severity metrics to incorporate risk characteristics and impact zones, forms the quantitative basis for the subsequent selection of risk response strategies. The study considers both the positive and negative dimensions of risks, as well as the interdependencies among them, during the process of selecting risk response strategies. Moreover, this paper tests and demonstrates the proposed optimization model using empirical data from a real-world liquefied petroleum gas (LPG) pipeline construction project. Consequently, this paper addresses two research questions: (1) What constitutes an appropriate framework for risk assessment and simultaneous selection of risk response strategies? (2) How can this framework be effectively validated? To address these research questions, this study integrates two distinct methodological components: (1) risk assessment using multi-criteria decision-making (MCDM) – specifically, fuzzy SWARA for weighting risk criteria and fuzzy WSM for evaluating and prioritizing risks. This stage produces a ranked list of risks with numerical scores but involves no optimization. (2) Risk response selection using mixed-integer linear programming (MILP) optimization – this stage formulates a genuine optimization problem (objective: minimize total risk value; constraints: budget, time, risk interdependencies, and overlapping responses). The MILP model is solved to optimality using CPLEX (specifically the Docplex library in Python) and constitutes the sole optimization component of the framework. The contribution lies in the integration of these two components within a single framework, not in claiming that every stage performs optimization.

**Table 3:** Summary of F-SWARA steps [79].

Step	Description
**One**	The criteria are presented, and the Decision-Makers (DMs) are asked to rank them based on their opinions, with the first criterion representing the highest importance and the last representing the least significant [79].
**Two**	Starting with the second criterion (*i* = 2), each DM is asked to express their opinion on the relative importance of criterion (*i*) to the previous criterion (*i* – 1). This is done using the scale presented in Table 4 [76].
**Three**	The opinions of the Decision-Makers (DMs) are aggregated by utilizing the minimum, arithmetic mean, and maximum values provided by each DM [79]: $\tilde{Ti}=\left({T_{i}}^{\left(l\right)},{T_{i}}^{\left(m\right)},{T_{i}}^{\left(u\right)}\right)\Rightarrow {T_{i}}^{\left(l\right)}=\underset{m}{\text{min}}\left\{{T_{i}}^{l}\right\};{T_{i}}^{\left(m\right)}=\frac{\text{1}}{m}\sum_{e=\text{1}}^{m}{T_{i}}^{(m)e};{T_{i}}^{\left(u\right)}=\underset{m}{\text{max}}\left\{{T_{i}}^{u}\right\}$ Where *m* is the number of DMs.
**Four**	Determine the ${\tilde{k}}_{i}$ and ${\tilde{q}}_{i}$ coefficients’ values using [79]: $\tilde{k_{i}}=\left\{ \begin{array}{c} \tilde{\text{1}}\;if\;i=\text{1}\\ \tilde{T_{i}}+\tilde{\text{1}}\;if\;i>\text{1} \end{array}\right.$ $\tilde{q_{i}}=\left\{ \begin{array}{c} \tilde{\text{1}}\;if\;i=\text{1}\\ \frac{\tilde{k_{i-\text{1}}}}{\tilde{k_{i}}}\;if\;i>\text{1} \end{array}\right.$
**Five**	Calculate the final weights using [79]: ${\tilde{w}}_{i}=\frac{\tilde{q_{i}}}{\sum_{j=\text{1}}^{m}\tilde{q_{j}}}$ where ${\tilde{w}}_{i}=\left({w_{i}}^{\left(l\right)},{w_{i}}^{\left(m\right)},{w_{i}}^{\left(u\right)}\right)$ represents the weight of criterion *i*.

Source: Adopted from Petrović et al. [79]

**Table 4:** Fuzzy linguistic scales [76].

Linguistic terms	Membership function
**Equally important**	(1,1,1)
**Moderately less important**	(2/3,1,3/2)
**Less important**	(2/5,1/2,2/3)
**Very little important**	(2/7,1/3, 2/5)
**Much less important**	(2/9,1/4, 2/7)

**Table 5:** Fuzzy linguistic terms for decision matrix values [81].

Linguistic terms for effects	Linguistic terms for probability	Percentage	Triple fuzzy numbers
**Negligible (N)**	Rare	(<1%)	(1,1,2)
**Very Low (VL)**	Very Unlikely	(1–5%)	(1,2,3)
**Low (L)**	Unlikely	(6–15%)	(2,3,4)
**Below Moderate (BM)**	Below Possible	(16–25%)	(3,4,5)
**Moderate (M)**	Possible	(26–40%)	(4,5,6)
**Above Moderate (AM)**	Above Possible	(41–60%)	(5,6,7)
**High (H)**	Likely	(61–75%)	(6,7,8)
**Very High (VH)**	Very Likely	(76–90%)	(7,8,9)
**Critical (C)**	Almost Certain	(>90%)	(8,9,9)

Unlike prior studies that focus either on risk assessment or on response selection in isolation, the proposed framework uniquely contributes by integrating both stages within a single optimization model, incorporating risk characteristics beyond probability and severity (detectability, manageability, predictability), explicitly modeling risk interdependencies and dual risk effects (threats and opportunities), and validating the model using empirical data from a real LPG pipeline construction project. The remainder of this paper is structured as follows. Section 2 reviews the relevant literature. Section 3 describes the methodologies employed. Section 4 tests the proposed integrated optimization approach using real-world data from an LPG pipeline construction project and discusses the findings. Section 5 presents the conclusions and policy recommendations.

## Background and literature review

Project Risk Management methodologies for risk analysis have been progressively developed since the early 2000s (e.g., [38,39]). Various project risk management models have been proposed since the 1990s to effectively manage project risks and enhance project success (e.g., [11,40]). The methodologies employed in these studies range from traditional techniques —such as Fault Tree Analysis [41], Event Tree Analysis [42], and the Risk Breakdown Structure (RBS) [43]—to more advanced methods, including decision-making approaches [44], Bayesian networks [45], and mathematical programming models [46].

To identify relevant studies for this review, we conducted a structured literature search in Scopus and Web of Science using the following keywords: “project risk assessment,” “risk response selection,” “optimization,” “MCDM,” “MILP,” “risk interdependency,” and “construction project.” The search was limited to peer-reviewed journal articles and conference papers published up to and including 2025. After screening titles and abstracts, we selected studies that explicitly addressed either risk assessment methods or risk response strategy selection in project management contexts. This review follows a scoping review approach, designed to map the range of methods used in project risk assessment and response selection rather than to evaluate the quality of individual studies.

To identify relevant resources and records pertaining to the research topic, a literature scoping process was undertaken, followed by an analysis of pertinent review articles (e.g., [8,30–32,34,35]). The scoping process involved searching the ScienceDirect and Web of Science databases for relevant titles and abstracts using the following keyword combinations: (1) Title, Abstract, and Keywords: (“Risk assessment” OR “Risk evaluation” OR “Risk analysis”) AND (“method” OR “tool” OR “approach” OR “model” OR “framework”); (2) Title, Abstract, and Keywords: (“Risk response” OR “Response selection” OR “Risk strategy”) AND (“method” OR “tool” OR “approach” OR “model” OR “framework”). Representative studies derived from this search are summarized in Tables 1 and 2, categorized by theme, methodological approach, and project context. A selection of recent risk assessment studies is summarized in Table 1.

The studies summarized in Table 1 [47–52] extend the earlier review papers [30–32] by focusing specifically on empirical applications in energy and construction projects. However, they reveal a shared limitation: they treat risk assessment and response selection as separate processes and rarely incorporate risk interdependencies or dual risk effects. By contrast, references [30–32] provide review-level overviews of project risk management and the risk management cycle, summarizing prior approaches to risk planning, assessment, and control. Although these review papers offer valuable bibliographic insights and identify current literature trends, they do not propose novel methods for risk assessment or risk response selection.

Similar to risk assessment, research on risk response strategies has a long history, with notable contributions from [53,54]. As previously mentioned, risk response methodologies can be categorized into four distinct groups: zonal-based methods (e.g., [54,55]), trade-off approaches (e.g., [48,56]), work breakdown structure (WBS)-based approaches (e.g., [33,57]), and optimization methods (e.g., [51, [58,59]). Additional relevant studies on project risk response strategies are presented in Table 2.

An analysis of recent literature reveals several important gaps in studies related to both risk assessment and the determination of risk response strategies. As summarized in Table 1, existing risk assessment studies largely overlook critical aspects such as risk characteristics, hybrid (compound) risk effects, and interdependencies among risks. In many cases, risks are evaluated in isolation, notwithstanding growing evidence that interactions among risks can significantly influence overall project performance.

Similarly, as shown in Table 2, studies focusing on risk response strategy selection predominantly rely on optimization-based approaches—including system dynamics optimization [64], MDCM-based optimization [62], and mathematical programming models—but often under restrictive or simplified assumptions [19,37,61]. These approaches typically neglect key considerations such as risk interdependencies, the simultaneous presence of positive and negative risks, and the dynamic nature of risk propagation across project stages. Moreover, many of these optimization models are developed for highly specific case contexts, which limits their generalizability and practical applicability.

More importantly, the literature tends to treat risk assessment and risk response selection as separate and sequential processes, analyzing them independently despite their inherent conceptual and functional interdependence within the risk management cycle. While optimization techniques are increasingly employed in response selection, they are rarely grounded in an integrated risk assessment framework. In contrast, this study contributes to the optimization literature by proposing an integrated framework that explicitly links risk assessment outputs to the optimization-based selection of risk response strategies. By embedding risk characteristics, interdependencies, and both positive and negative risk effects into a unified decision-support structure, the proposed framework addresses key limitations in existing studies and enhances the effectiveness of risk-informed optimization in project risk management. While the literature offers a variety of optimization techniques for either risk assessment or response selection, no single framework integrates both stages while simultaneously accounting for risk characteristics beyond probability and severity, dual risk effects (threats and opportunities), and risk interdependencies. This gap directly motivates the design of our framework presented in Figs 3 and 4. The framework proposed in this study is designed to fill the gap.

The incremental contribution of this study is threefold: (1) a structured integration of risk characteristics (probability, detectability, manageability) and impact zones into a fuzzy MCDM assessment; (2) the explicit modeling of risk interdependencies and overlapping responses within a MILP optimization for response selection; and (3) a real-world LPG pipeline case study demonstrating the combined approach. This is not a claim of a new optimization algorithm, but rather a domain-specific application and integration of existing methods (F SWARA, F WSM, MILP) tailored to LPG pipeline projects.

## Methods

To address the research objective of developing an approach for risk assessment and response strategy selection, a multi-stage model is proposed. This model systematically addresses risk identification and planning, risk analysis and evaluation, and the selection of risk response strategies. An outline of the model is presented in Fig 4. The proposed framework departs from a standard Fuzzy SWARA application in four fundamental ways. First, while standard Fuzzy SWARA is used solely for criteria weighting, our framework integrates it with Fuzzy WSM for risk scoring and with MILP for response selection, creating an end-to-end decision support system. Second, the framework incorporates risk interdependencies (Table 6) and dual risk effects (threats and opportunities), which are absent from conventional Fuzzy SWARA implementations. Third, the framework explicitly links risk assessment outputs (risk scores) to response selection constraints (budget, time, risk tolerance), as shown in Eqs. (6)–(16). Fourth, the framework is validated on empirical data from a real-world LPG pipeline project, demonstrating practical applicability beyond methodological illustration. The design choices reflected in Fig 3 (risk properties and process phases) are directly operationalized in Fig 4’s three-stage structure: Stage 1 gathers the risk properties, Stage 2 evaluates them using fuzzy MCDM, and Stage 3 selects responses under constraints. Collectively, these features justify the use of the term “framework” to describe our contribution.

**Table 6:** Risk interdependencies.

Source	Target	Threshold	Effect
**R01**	R04	R01 (C1) > 30%	R04 (C02) dec by 10%
**R05**	R11	R05 (C1) > 30%	R11 (C06) dec by 25%
**R06**	R15	R06 (C2) > 50%	R15 (C05) inc by 10%
		R06 (C2) > 50%	R15 (C06) dec by 10%
	R07	R06 (C2) > 50%	R07 (C03) dec by 15%
**R17**	R04	R17 (C4) > 20%	R04 (C01) dec by 20%
	R09	R17 (C4) > 20%	R09 (C01) dec by 20%
**R18**	R05	R18 (C1) > 40%	R05 (C04) inc by 15%

Source: Research findings.

As illustrated in Fig 4, the proposed framework comprises three sequential stages: risk identification, risk evaluation, and risk response selection. Each stage is discussed in detail in the following three sections. Section 3.1 addresses the first stage of the framework. After evaluating alternative approaches, we determined that the Delphi method is the most suitable technique for risk identification in this study. Section 3.2 describes the second stage of the framework, which focuses on the evaluation of identified risks. We propose a MCDM approach to weigh the risk characteristics and subsequently calculate a score for each risk. Section 3.2 is divided into two subsections. The first subsection presents the weighting of risk characteristics using the fuzzy SWARA method. The second subsection presents the risk evaluation using the fuzzy WSM model. The third and final stage focuses on selecting appropriate risk response strategies. We propose a MILP model to identify the optimal set of responses that minimizes the impact of identified risks, while respecting project-specific constraints (e.g., budget, resources, and time). Although Fig 4 presents the framework as a logical workflow, its implementation is fully computational. Stage 2 (F-SWARA and F-WSM) is executed via a Python script that automates the fuzzy aggregation, weighting, and defuzzification calculations described in Sections 3.2 (risk assessment sub-section). Stage 3 is solved using the CPLEX optimization engine (via the Docplex library in Python), which performs mixed-integer linear programming with the constraints and variables defined in Table 7 and Eqs. (6)–(16). This automation distinguishes the proposed framework from a manual spreadsheet approach.

**Table 7:** Sets, parameters and decision variables of the model.

	Sets
*R*	Set of risks
*P*	Set of risk criteria (characteristics & impact zones)
*S*	Set of risk response strategies
*I(i)*	Set of interdependencies of risk *i*
	**Decision variables**
$s_{k}$	Binary variable: whether risk response strategy k is executed or not
$y_{ij}^{k}$	Binary variable; whether risk response *k* affects risk criteria *j* of project *i* or not
$\theta_{ij}^{i^{\prime }j^{\prime }}$	Binary variable; whether the risk interdependency of criteria *j* of project *i* and criteria $j^{\prime }$ of risk $i^{\prime }$ is established or not
	**Parameters**
$V_{i}$	Risk score of risk *i*
$w_{j}$	Weight of risk criteria *j*
$x_{ij}$	The value of criteria *j* of risk *i*
$c_{i}$	Financial impact of risk *i*
$d_{i}$	Delay impact of risk *i*
$q_{i}$	Quality impact of risk *i*
C	Financial tolerance
D	Delay tolerance
Q	Tolerance to decline in quality
$b_{k}$	Cost to implement risk response strategy *k*
***B***	Total budget planned for risk prevention
$t_{k}$	Time required to implement risk response *k*
***T***	Total float time available for risk response strategy implementation
$e_{ij}^{k}$	The effect of risk response strategy *k* on the *j* criteria of risk *i*
$\gamma_{kk^{\prime }}$	Binary parameter: whether risk response strategies *k* and $k^{\prime }$ overlap or not
$\lambda_{ij}^{i^{\prime }j^{\prime }}$	Interdependency threshold of criteria *j* of project *i* and criteria $j^{\prime }$ of risk$i^{\prime }$
$\delta_{ij}^{i^{\prime }j^{\prime }}$	Percent of change to risk criteria *j* of project *i* due to the interdependency with risk criteria $j^{\prime }$ of risk$i^{\prime }$
M	A big number
ε	Small number

Source: Research findings.

### Risk planning and identification

The first stage involves gathering essential data for subsequent stages including comprehensive list of potential risks (both threats and opportunities), their impacts and characteristics, risk interdependencies, and project-related information (e.g., allocated project budget and timeline for implementing risk response strategies). This information can be obtained from two primary sources: existing literature and lessons learned from previous projects, in addition to insights from specialists and experts.

Data collection can be conducted using various methods. This study proposes an initial step consisting of a critical review of the literature and relevant risk cases [65] to establish a foundational dataset. Subsequently, expert opinions should be employed to refine and contextualize the data for the specific project. Two approaches are recommended for this refinement: the checklist method [25], which yields faster and simpler results, and the Delphi method [27], which produces more precise outcomes but requires a greater time investment.

This stage aims to gather key information for subsequent stages, including risk characteristics (e.g., probability, predictability; uniqueness) and their impact zones (cost, time, scope, resources); a comprehensive list of risks with brief definitions highlighting their characteristics and impacts; risk interdependencies and interactions; feasible response strategies; and project information (e.g., budget and available float time). Although checklists or questionnaires may be suitable for certain purposes, the Delphi method is more appropriate when finer details are required.

### Risk assessment

The risk assessment stage comprises two primary processes: first, the determination of the weight for each risk characteristic and impact zone; and second, the evaluation of each risk based on these characteristics and impact zones. The first process employs a suitable weighting method whereas the second utilizes a Multi-Criteria Decision-Making (MCDM) method. This MCDM method evaluates each risk according to the weights and values of the respective impacts and characteristics, yielding a ranked list of risks accompanied by numerical risk scores that indicate their relative significance. As previously noted, risk analysis typically encompasses two approaches: quantitative and qualitative. Nevertheless, an emerging trend in risk analysis research involves the incorporation of fuzzy logic (e.g., [66,67]). Accordingly, this study integrates fuzzy logic into both the analysis and evaluation stages. The selection of Fuzzy SWARA for criterion weighting, Fuzzy WSM for risk evaluation, and Mixed-Integer Linear Programming (MILP) for response strategy selection was guided by four explicit criteria: (i) compatibility with limited and uncertain expert input; (ii) preservation of linearity for seamless integration with MILP; (iii) transparency and ease of validation by project stakeholders; and (iv) computational efficiency for real-world project application.

Several alternative methods were systematically evaluated against these criteria and deliberately excluded. Fuzzy Analytic Hierarchy Process (F-AHP) [21], while well-established and capable of handling hierarchical structures, requires an excessive number of pairwise comparisons. For six criteria, F-AHP demands 15 comparisons per expert, whereas Fuzzy SWARA requires only five, leading to expert fatigue given the study’s 20 risks and three experts. Fuzzy TOPSIS and WASPAS [19,37,49,51,68–70] offer advantages in ranking accuracy but rely on non-linear normalization and distance calculations. Incorporating them would transform the subsequent optimization into a non-linear or mixed-integer non-linear problem (MINLP), substantially increasing computational complexity without commensurate benefit. Bayesian networks [45] are powerful for probabilistic risk dependency modeling but require conditional probability tables that were unavailable from project experts. Heuristic and genetic algorithms [46,51] could solve the response selection problem; however, they do not guarantee global optimality—a critical requirement for this study’s optimization stage. In contrast, MILP solved with CPLEX (specifically the Docplex library in Python) guarantees global optimality within the formulated constraints.

Consequently, the integrated framework combining F-SWARA, F-WSM, and MILP is the most appropriate choice given the available data and the problem structure. It offers a linear, transparent, and expert-friendly approach that ensures global optimality, albeit with reduced capability for modeling non-linear interactions. Overall, the selected methodology strikes an optimal balance among methodological rigor, data availability, computational tractability, and decision-maker transparency for the LPG pipeline case and similar construction projects. The weighting and MCDM methods employed in this study are discussed in the following two sections.

#### Fuzzy SWARA.

Given the specific context of the problem— characterized by limited data availability and the essential role of expert opinion—a subjective weighting method is more suitable. The Step-wise Weight Assessment Ratio Analysis (SWARA) is a Multi-Criteria Decision-Making (MCDM) technique that prioritizes criteria based on expert judgment, thereby incorporating the relative importance of each criterion. SWARA was originally introduced by Keršulienė et al. [71] and has been employed in various studies (e.g., [72,73]).

Several Multi-Criteria Decision-Making (MCDM) methods exist for criteria weighting in project risk management, including the Analytic Hierarchy Process (AHP) [21], TOPSIS [49], VIKOR, the Best Worst Method (BWM) [74], and the Level Based Weight Assessment (LBWA) method [75]. However, the fuzzy Step-wise Weight Assessment Ratio Analysis (F-SWARA) was selected for this study for several reasons. First, SWARA offers lower computational complexity and requires fewer pairwise comparisons. Unlike AHP, which requires n(n − 1)/2 comparisons, SWARA requires only n − 1 comparisons, significantly reducing the cognitive burden on experts [71]. Compared to BWM and LBWA, SWARA captures expert preferences in fewer steps, thus requiring less time [74,75]. Second, unlike TOPSIS and VIKOR, which are primarily designed for alternative ranking, SWARA is specifically suited for deriving criterion weights from expert judgments. Third, SWARA allows experts to express relative importance using intuitive linguistic terms (e.g., “moderately less important”) rather than numerical ratio scales, which is more practical in fuzzy, data-limited environments [76]. Fourth, SWARA’s demonstrated synergy with the Weighted Sum Model (WSM) [77,78] maintains linearity in our subsequent optimization stage (Section 3.3). Finally, when the number of criteria is relatively small, SWARA achieves accuracy comparable to more complex methods. While fuzzy AHP is an alternative, SWARA’s lower computational complexity and fewer comparisons make it more practical for time-constrained expert panels, as in the present case study. In addition, fuzzy SWARA (F-SWARA) has been applied in various research contexts. This study follows the steps outlined in [79].

In addition, fuzzy SWARA (F-SWARA) has been applied in various research contexts. This study follows the steps outlined by Petrović et al. [79], summarized in Table 3, which utilize the linguistic terms discussed by Zarbakhshnia et al. [76], presented in Table 4. All equations presented in Table 3 are adapted from [79] without modification, as the steps are standard for F-SWARA implementation.

#### Fuzzy WSM.

Once the weights of the risk characteristics have been determined, the next step is to establish the decision matrix and evaluate each risk. This evaluation is performed using a Multi-Criteria Decision-Making (MCDM) method [80]. The Weighted Sum Model (WSM), also known as the Simple Additive Weighting (SAW), is a fundamental MCDM technique, with origins tracing back to the early work of Churchman & Ackoff [68]. Although rarely used in contemporary applications, it is often combined with the Weighted Product Model (WPM) to form the Weighted Aggregated Sum Product Assessment (WASPAS) method [69]. The fuzzy WSM follows steps similar to those of fuzzy WASPAS [70]. Like SWARA, WSM was chosen for its simplicity and straightforwardness in determining the value of each risk alternative. The selection of the WSM method over more computationally complex alternatives, such as WASPAS, is primarily driven by the requirements of the subsequent mathematical programming stage. WSM was employed to maintain a linear model structure, thereby avoiding the complexities inherent in non-linear formulations. Furthermore, the efficacy of the WSM-SWARA integration is well-documented in existing literature, which demonstrates significant synergy between these methods [77,78].

The fuzzy WSM (F-WSM) involves the following steps:

**Step 1.** The decision matrix is established using Eq. (1), with *a* risk and *c* risk criteria. Based on expert opinion, the decision-makers (DMs) evaluate each risk against each criterion using a scale from 1 to 9. These evaluations are then converted into triangular fuzzy numbers using the linguistic scale proposed by Tesfamariam and Sadiq [81], as shown in Table 5.


$$\begin{array}{c} \tilde{X}=[\begin{array}{ccc} \begin{array}{cc} {\tilde{x}}_{\text{1}\text{1}} & {\tilde{x}}_{\text{1}\text{2}} \end{array} & \cdots & {\tilde{x}}_{\text{1}c}\\ \begin{array}{cc} {\tilde{x}}_{\text{2}\text{1}} & {\tilde{x}}_{\text{2}\text{2}} \end{array} & \cdots & {\tilde{x}}_{\text{2}c}\\ \begin{array}{cc} \begin{array}{c} \vdots \\ {\tilde{x}}_{a\text{1}} \end{array} & \begin{array}{c} \vdots \\ {\tilde{x}}_{a\text{2}} \end{array} \end{array} & \begin{array}{c} \ddots \\ \ldots \end{array} & \begin{array}{c} \vdots \\ {\tilde{x}}_{ac} \end{array} \end{array}],\text{1}\leq i\leq a;\text{1}\leq j\leq c \end{array}$$ (1)

**Step 2.** The opinions of the decision-makers are aggregated when more than one DM is involved (m>1). For this purpose, the fuzzy Heronian operator proposed by Yu [82] is used to determine the final fuzzy value, as outlined in Eq. (2). Here, *p* and *q* are sets of non-negative numbers (p,q≥0). The operator is taken directly from [82] without modification.


$$\begin{array}{c} {\tilde{x}}_{ij}=\left({\tilde{x}}_{ij}^{l},{\tilde{x}}_{ij}^{m},{\tilde{x}}_{ij}^{u}\right)=\left\{ \begin{array}{c} {\tilde{x}}_{ij}^{l}={\left(\frac{\text{2}}{m(m+\text{1})}\sum_{i=\text{1}}^{a}\sum_{j=i}^{c}{\tilde{x}}_{i}^{lp}{\tilde{x}}_{j}^{lq}\right)}^{\frac{\text{1}}{p+q}}\\ {\tilde{x}}_{ij}^{m}={\left(\frac{\text{2}}{m(m+\text{1})}\sum_{i=\text{1}}^{a}\sum_{j=i}^{c}{\tilde{x}}_{i}^{mp}{\tilde{x}}_{j}^{mq}\right)}^{\frac{\text{1}}{p+q}}\\ {\tilde{x}}_{ij}^{u}={\left(\frac{\text{2}}{m(m+\text{1})}\sum_{i=\text{1}}^{a}\sum_{j=i}^{c}{\tilde{x}}_{i}^{up}{\tilde{x}}_{j}^{uq}\right)}^{\frac{\text{1}}{p+q}} \end{array}\right. \end{array}$$ (2)

**Step 3.** The weighted matrix is then determined using Eq. (3)[68]:


$$\begin{array}{c} {\overset{―}{{\tilde{x}}_{ij}}=\tilde{x}}_{ij}\tilde{w_{j}} \end{array}$$ (3)

where $\tilde{w_{j}}$ denotes the weight of the corresponding criterion, as determined in the second stage.

**Step 4.** Finally, the scores of each risk are calculated using Eq. (4) by Churchman and Ackoff [68], which sums the weighted values across each row of the decision matrix.


$$\begin{array}{c} \tilde{K_{i}}=\sum_{j}^{n}\overset{―}{{\tilde{x}}_{ij}}\;,i=[\text{1},c] \end{array}$$ (4)

To de-fuzzify the final score $\tilde{K_{i}}$, Eq. (5) is used by Keshavarz Ghorabaee et al. [70]:


$$\begin{array}{c} K_{i}=\frac{\text{1}}{\text{3}}\left(K_{il}+K_{im}+K_{iu}\right) \end{array}$$ (5)

Equations (3)– (5) are standard weighted sum and defuzzification calculations used in fuzzy MCDM [68,70] and have not been modified. All equations in this section are numbered sequentially (1)– (5). Equations (6)– (16), presented in the next section, are original contributions of this study, developed specifically for the risk response selection problem. These equation numbers are used consistently throughout the manuscript.

### Selection of risk response strategies

Once a comprehensive list of risks and risk response strategies has been compiled in the first stage, and the risk values have been determined in the second stage, the third stage uses this information to select an optimal set of risk responses through mixed-integer linear programming. Unlike the risk planning, identification, and assessment stages — which employ MCDM without optimization — the third stage formulates a genuine optimization problem. In this study, ‘optimization’ is defined as the deterministic selection of a subset of risk response strategies that minimizes total project risk value subject to constraints on budget, time, risk interdependencies, and response overlaps. This constitutes a binary linear combinatorial optimization problem (mixed-integer linear programming, MILP), not a heuristic or MCDM ranking. The objective function (Eq. 6) is linear in the decision variables, and constraints (Eq. 8–16) include binary variables, linear equalities, and inequalities. The problem is solved to optimality using CPLEX. Accordingly, optimization herein refers to the systematic, solver-based identification of the globally optimal combination of response strategies to minimize residual risk while satisfying all project constraints. The goal is to optimize the risk values by enhancing positive risks and reducing negative risks through the selection of appropriate responses, subject to the project’s constraints. The variables, sets, and parameters are defined in Table 7. The mathematical optimization model, comprising Eqs. (6) – (16), is structured as follows:

#### Optimization problem formulation.

The optimization problem formulated in this section addresses the following decision problem: Given a set of identified risks R with associated scores $V_{i}$, a set of candidate risk response strategies S with implementation costs $b_{k}$ and times $t_{k}$, and project constraints —namely, budget *B*, float time *T*, and risk tolerances for cost *C*, delay *D*, and quality *Q*. The objective is to select a subset of response strategies that minimizes the total risk value while satisfying all constraints.


$$\begin{array}{c} \text{Min}(z)=\sum_{i}V_{i}\;i\in R \end{array}$$ (6)

$$\begin{array}{c} V_{i}=\frac{\left({V_{i}}^{\left(l\right)}+{V_{i}}^{\left(m\right)}+{V_{i}}^{\left(u\right)}\right)}{\text{3}}\;\&{\tilde{V}}_{i}=\sum_{j}^{P}{(\tilde{x}}_{ij}-\sum_{k}{\tilde{y}}_{ij}^{k}{\tilde{e}}_{ij}^{k}+{\tilde{\delta }}_{ij}^{i^{\prime }j^{\prime }}\theta_{ij}^{i^{\prime }j^{\prime }}){\tilde{w}}_{j}\;i\in R\&j\in P\&k\in S\&i^{\prime }j^{\prime }\in I(i) \end{array}$$ (7)

s.t.


$$\begin{array}{c} \sum i\sum jy_{ij}^{k}\geq s_{k}k\in S \end{array}$$ (8)

$$\begin{array}{c} s_{k}+s_{k^{\prime }}+\gamma_{kk^{\prime }}\leq \text{2}k^{\prime }\&k\in S \end{array}$$ (9)

$$\begin{array}{c} \sum i{(c}_{i}-\sum_{k}{y_{ij}^{k}e}_{ifinancial}^{k}+{c_{i}\delta }_{ij}^{i^{\prime }j^{\prime }}\theta_{ij}^{i^{\prime }j^{\prime }})\leq C\;i\in R\&k\in S\&i^{\prime }j^{\prime }\in I(i) \end{array}$$ (10)

$$\begin{array}{c} \sum i{(d}_{i}-\sum_{k}{y_{ij}^{k}e}_{idelay}^{k}+{di\delta }_{ij}^{i^{\prime }j^{\prime }}\theta_{ij}^{i^{\prime }j^{\prime }})\leq D\;i\in R\&k\in S\&i^{\prime }j^{\prime }\in I(i) \end{array}$$ (11)

$$\begin{array}{c} \sum i{(q}_{i}-\sum_{k}{y_{ij}^{k}e}_{iquality}^{k}+q_{i}\delta_{ij}^{i^{\prime }j^{\prime }}\theta_{ij}^{i^{\prime }j^{\prime }})\leq Q\;i\in R\&k\in S\&i^{\prime }j^{\prime }\in I(i) \end{array}$$ (12)

$$\begin{array}{c} \sum_{k}b_{k}s_{k}\geq B\;k\in S \end{array}$$ (13)

$$\begin{array}{c} \sum_{k}t_{k}s_{k}\geq T\;k\in S \end{array}$$ (14)

$$\begin{array}{c} {(\tilde{x}}_{ij}-{\tilde{y}}_{ij}^{k}{\tilde{e}}_{ij}^{k})-\lambda_{ij}^{i^{\prime }j^{\prime }}+\varepsilon \leq M\theta_{ij}^{i^{\prime }j^{\prime }}\;i\in R\&j\in P\&k\in S\&i^{\prime }j^{\prime }\in I(i) \end{array}$$ (15)

$$\begin{array}{c} {(\tilde{x}}_{ij}-{\tilde{y}}_{ij}^{k}{\tilde{e}}_{ij}^{k})-\lambda_{ij}^{i^{\prime }j^{\prime }}\geq M(\text{1}-\theta_{ij}^{i^{\prime }j^{\prime }})\;i\in R\&j\in P\&k\in S\&i^{\prime }j^{\prime }\in I(i) \end{array}$$ (16)

Eq. (6) represents the objective function of the optimization problem, which minimizes the total risk value, defined as $\text{z}=\sum_{i}V_{i}$. This objective aggregates the risk scores of all identified risks. Positive risks (opportunities) contribute negatively to $V_{i}$, while negative risks (threats) contribute positively. Consequently, minimizing the sum encourages exploiting opportunities and the mitigation of threats. It is important to note that the values of threats and opportunities effectively negate each other. The fuzzification of data follows the linguistic terms outlined in Table 5. The fuzzification of data follows the linguistic terms outlined in Table 5. The resulting values are derived from the F‑WSM model and must be transformed into crisp values, as shown in Eq. (7). Risk values are influenced by risk characteristics, which are in turn affected by risk response strategies and interdependencies among risks. The weights of the risk characteristics are calculated using the F-SWARA method.

**Decision variables:** The binary variable ${\text{s}}_{k}$indicates whether response strategy k is selected (1) or not (0). The auxiliary binary variables${(y}_{ij)}^{k}$ and${(\theta }_{ij}^{i^{\prime }j^{\prime }})$ capture, respectively, whether a given response affects a specific risk criterion and whether a given risk interdependency is activated.

**Constraints:**

- Eq. (8): A response strategy can affect a risk criterion only if it is selected. In the model, a utility variable$y_{ij}^{k}$ is introduced to represent the possible effects of selecting risk response strategies $s_{k}$. The relationship between these two is expressed in Eq. (8). Eq. (9): Overlapping responses ${(\gamma }_{kk^{\prime }}$=1) cannot be selected simultaneously. This constraint ensures that two or more responses that overlap (e.g., share resources or conflict in implementation) are not chosen together. Eqs. (10–12): The total residual risk impacts on cost, delay, and quality must remain within project tolerances. These equations represent the tolerance of the project’s main constraints, including quantitative values for delays and costs, as well as a qualitative scale for overall project quality.
- Eqs. (13–14): The total cost and time of selected responses must not exceed the budget *B* and float time *T*. These equations depict the allocated budget and float time for implementing risk responses.
- Eqs. (15–16): Risk interdependencies are activated when a risk criterion exceeds a threshold $\left(\lambda_{ij}^{i^{\prime }j^{\prime }}\right)$, triggering a percentage change${(\delta }_{ij}^{i^{\prime }j^{\prime }})$in a related risk criterion. Finally, these equations illustrate that if the current value of a specific criterion of a risk exceeds a certain threshold, it will affect the characteristics of a related risk by a certain percentage.

**Optimality:** The problem is formulated as a mixed-integer linear program (MILP). Due to the linearity of the objective function and constraints, the CPLEX solver, which employs a branch-and-cut algorithm, guarantees global optimality (or a proof of infeasibility) for the formulated problem. Following this model, a set of risk response strategies is selected to maximize the exploitation of opportunities and minimize threats, while respecting the project’s natural constraints. All variables, sets, and parameters in Table 7 are original to this study. The mathematical programming formulation (Eqs. 6–16) is adapted from standard MILP conventions, but the model’s distinctive structure —including risk interdependencies, dual risk effects, and response overlap constraints— represents a novel contribution.

The organizational data used in this case study were obtained from internal project reports and documentation related to a liquefied petroleum gas (LPG) pipeline construction project, supplied by a petroleum refinery company. This study did not involve human participants, medical records, or any form of identifiable private information. Therefore, informed consent was neither required nor obtained. To protect the confidentiality of the originating organization, all data have been fully anonymized, and no identifiable information is disclosed in this manuscript. The findings are presented solely for methodological illustration and should not be interpreted as an evaluation of the specific organization’s practices or performance.

## Empirical results and discussion: An illustrative case study

### Case study design

To evaluate the proposed framework, a single-case study design was adopted, focusing on a liquefied petroleum gas (LPG) pipeline construction project. The project involved constructing a 120 km pipeline connecting an oil refinery to petrochemical complexes in another city, utilizing 16-inch steel pipes (API-5L), and also served to connect LPG flow between two cities. The project duration was 18 months (mid-2023 to late 2024). The case was selected due to its typical characteristics for pipeline construction projects in the region, making it suitable for illustrative testing of the framework.

Data were collected from two primary sources: (1) official project documentation, including risk registers, progress reports, post-project reviews, and lessons learned reports; and (2) expert elicitation. Risk identification followed a multi-method approach. First, a systematic literature review of pipeline construction risks was conducted to develop an initial risk checklist. Second, this checklist was reviewed and refined through three rounds of the Delphi method with three domain experts. The Delphi process involved: (a) independent expert ranking and commenting on risks; (b) aggregation and anonymized feedback; and (c) a second round for convergence. The experts were also questioned using checklists to identify risks and were interviewed following the three Delphi rounds to obtain other information (e.g., regarding risk responses).

To gather additional risk-related information, three experts associated with the project were consulted for their insights and relevant risk data. These experts were purposively selected based on the following criteria: (i) at least ten years of experience in oil and gas pipeline construction projects; (ii) direct involvement in the focal LPG pipeline project; and (iii) expertise spanning project management, risk analysis, and engineering. Specifically, the expert panel consisted of two project managers (each with more than 20 years of experience) and one executive supervisor (with more than 10 years of experience). We acknowledge that this is a small panel; therefore, the results should be interpreted as a proof-of-concept rather than a statistically generalizable finding. The primary purpose here is to demonstrate the operational feasibility of the integrated approach, not to claim robust population-level validity. Risk data were collected corresponding to the project’s planning and early execution phases. Expert elicitation sessions (each lasting 60–90 minutes) were conducted virtually and recorded with consent.

Because the project had ended, it was difficult to contact lower operational managers; however, the main project managers and the executive supervisor were successfully contacted. No additional stakeholders (e.g., contractors, local authorities) were directly consulted due to data access restrictions —a limitation we acknowledge. Subsequently, the study implements each stage of the proposed model and presents the findings from each stage to evaluate the model.

#### Data processing and preparation.

Raw data from project documentation (risk registers, progress reports, lessons-learned reports) were transcribed into structured Excel spreadsheets. Expert elicitation data were collected via structured questionnaires and Delphi rounds. All qualitative risk assessments (e.g., linguistic terms such as “-High,”- “-Low,”- “-Critical”-) were converted into triangular fuzzy numbers using the scales in Tables 4 and 5. The fuzzy decision matrix (Table A1) was constructed by aggregating individual expert judgments using the fuzzy Heronian operator (Equation 2). Defuzzified risk scores (Equation 5) were then used as inputs to the MILP model. No data imputation or transformation beyond fuzzification and defuzzification was performed.

### Experimental design and procedure

The experimental procedure follows the three-stage framework illustrated in Fig 4. Below, we detail the sequential steps, including iterations and validation checks.

Step 1: Raw risk data from project documentation and expert inputs were transcribed into structured formats, as presented in Tables 6,8,9. Risks were categorized as threats or opportunities based on expert consensus, with 100 percent agreement achieved after three Delphi rounds.

**Table 8:** List of identified risks.

Risk	Definition
**R01** ^**-**^	Unexpected geological conditions
**R02** ^**-**^	Environmental obstacles along the route
**R03** ^**-**^	Delays due to adverse weather conditions
**R04** ^**-**^	Technical issues and design errors
**R05** ^**-**^	Delays in raw material supply and low quality
**R06** ^**-**^	Labor shortages or worker strikes
**R07** ^**-**^	Additional costs due to inaccurate cost estimation
**R08** ^**-**^	Accidents and incidents at the construction site
**R09** ^**-**^	Defects and failures of heavy construction equipment
**R10** ^**-**^	Soil instability and erosion in areas adjacent to the pipeline
**R11** ^**-**^	Welding defects and connection failures
**R12** ^**-**^	Premature pipe corrosion during construction
**R13** ^**-**^	Land use rights issues and legal problems
**R14** ^**-**^	Delays in obtaining construction permits
**R15** ^**-**^	Lack of coordination and inadequate project management
**R16** ^**+**^	Accelerated progress than initially planned and cost reduction
**R17** ^**+**^	Technological advancements and reduced construction time
**R18** ^**+**^	Cost savings through bulk material purchases
**R19** ^**+**^	Development of local infrastructure to facilitate execution
**R20** ^**+**^	Better-than-expected soil conditions and reduced construction complexity

Source: Research findings.

**Table 9:** Available risk response strategy options.

Strategy	Definition
**S01**	Geotechnical Investigation & Adaptive Design
**S02**	Measures for Mitigating Environmental Impact
**S03**	Real-Time On-Site Weather Monitoring
**S04**	Quality Control & Corrosion Prevention Measures
**S05**	Comprehensive Supply Chain Management with Diverse Sourcing Solutions
**S06**	Labor Contracting Solutions and Workforce Development Programs
**S07**	Enhanced Cost Estimation and Strategic Contingency Planning
**S08**	Establishing Site Safety Protocols and Comprehensive Training
**S09**	Preventive Maintenance policies
**S10**	Investments in the legal team and claims management
**S11**	Local partnership programs
**S12**	Investment in the R&D team and Supporting Technology Sub-Projects

Source: Research findings.

Step 2: The fuzzy SWARA (F-SWARA) method was employed for criteria weighting. Three experts independently assigned linguistic ratings (see Table 4) to pairwise criteria comparisons. The fuzzy Heronian operator (Equation 2) was used to aggregate individual judgments. Convergence, defined as less than a 10 percent change in weights between rounds, was achieved after three Delphi rounds.

Step 3: The fuzzy weighted sum model (F-WSM) was applied for risk scoring. Each expert evaluated all 20 risks against six criteria using the linguistic scale presented in Table 5. The fuzzy Heronian operator aggregated the individual scores. A consistency check was performed by comparing individual risk rankings. Spearman’s rank correlation coefficients between experts ranged from 0.82 to 0.91, indicating acceptable agreement.

Step 4: The mixed-integer linear programming (MILP) model was employed to select optimal risk response strategies. The optimization model, formally defined in Equations 6–16, was solved using the CPLEX optimization engine via the Docplex library in Python. A baseline scenario without any response strategies was run for comparison. To validate robustness, sensitivity analyses were conducted, including 21 scenarios for risk criteria values and 11 scenarios for response requirements. These results are presented in Section 4, Fig 11.

Step 5: The results of the optimization model were presented to the three experts for face validation. All experts confirmed that the selected response strategies (S02, S05, S07, S09, S11, and S12) were practically feasible and aligned with project constraints. No further iterations were required, as documented in Section 4.

#### Parameter justification.

The numerical values employed in the optimization model originate from three primary sources. First, risk scores and criteria values are derived directly from the fuzzy decision matrix (Table A1), which is grounded in expert elicitation and project documentation. Second, response costs and time requirements (Table A3) are obtained from the project’s actual cost estimates and construction schedule. Third, the budget constraint (B=8 million) and float time (T=5 months) reflect the project’s documented tolerances. The criteria weights (W=[0.244,0.043,0.076,0.487,0.122,0.029]) are drawn from the F-SWARA analysis (Table 10). All remaining parameters—such as the risk interdependency thresholds presented in Table 6—are based on expert consensus achieved through the Delphi process. Importantly, no arbitrary or empirically selected values are employed without explicit justification.

**Table 10:** Weights of risk criteria based on the F-SWARA method.

		S			K			q		W
**C4**	–	–	–	1	1	1	1	1	1	(0.381, 0.487, 0.601)
**C1**	0.667	1	1.5	1.667	2	2.5	0.4	0.5	0.6	(0.152, 0.244, 0.36)
**C5**	0.667	1	1.5	1.667	2	2.5	0.16	0.25	0.36	(0.061, 0.122, 0.216)
**C3**	0.286	0.611	1.5	1.286	1.611	2.5	0.064	0.155	0.28	(0.024, 0.076, 0.168)
**C2**	0.286	0.778	1.5	1.286	1.778	2.5	0.026	0.087	0.218	(0.01, 0.043, 0.131)
**C6**	0.286	0.444	0.67	1.286	1.444	1.667	0.015	0.06	0.169	(0.006, 0.029, 0.102)

Source: Research findings.

### Handling of expert bias and subjectivity

The use of only three experts is a limitation; however, several measures were implemented to mitigate potential bias and enhance reliability.

1. The Delphi method for consensus building. Three iterative rounds with anonymized feedback reduced the influence of dominant individuals and encouraged convergence. After the third round, the coefficient of variation for risk criteria weights fell below 0.15, indicating acceptable consensus; 2. fuzzy logic to incorporate uncertainty. Triangular fuzzy numbers (Tables 4 and 5) explicitly represent the vagueness inherent in expert judgments, rather than forcing precise point estimates. The fuzzy Heronian operator (Equation 2) aggregates opinions without discarding lower and upper bounds; 3. inter-rater reliability assessment. Spearman’s rank correlation coefficients for risk rankings (Step 3) were calculated pairwise, yielding the following results: ρ (E1, E2) = 0.85, ρ (E1, E3) = 0.82, and ρ (E2, E3) = 0.91 (p < 0.01 for all). According to standard thresholds (0.80–1.00), these values indicate “-strong”- to “-very strong”- agreement; 4. triangulation with documentary evidence. Expert inputs were cross-validated against official project documentation (risk registers, incident reports) where available. For 16 out of 20 risks, documentary evidence corroborated expert assessments. Discrepancies were discussed during the third Delphi round and subsequently resolved; and 5. Sensitivity analysis. As shown in Fig 11, moderate variations (±30 percent) in input parameters did not alter the main conclusions. Selected response strategies changed only when response requirements were reduced by more than 40 percent, suggesting that the results are not overly sensitive to individual expert judgments.

Finally, regarding the justification for the relatively small sample size (n = 3), we recognize that larger panels are generally preferable. However, the Delphi literature indicates that three to five experts may be sufficient when they possess deep domain knowledge and the problem scope is narrowly defined [27,65]. Given the specialized nature of LPG pipeline construction and the seniority of the selected experts (with 15–22 years of experience), three experts were considered sufficient for this illustrative case study. Nonetheless, we acknowledge this as a limitation and recommend the inclusion of larger expert panels in future research.

Initially, we conducted a literature review on pipeline construction projects to develop a checklist of potential risks, covering both threats and opportunities. This checklist was then distributed to experts to gather their opinions and identify the critical risks affecting the project. Based on the checklist results, the final list of risks is presented in Table 8.

The identified risks encompass both threats and opportunities, as indicated by the positive and negative superscript indicators in the first column of Table 8. These risks are thematically grouped into four categories:

a) On-site condition risks: R01, R02, R03, R10, R20
b) Resource consumption risks: R05, R06, R18, R19
c) Technical and technological risks: R04, R08, R09, R11, R12, R17
d) Management‑related risks: R07, R13, R14, R15, R16

Based on expert opinions and the existing documentation, the risk characteristics of the risks and their impact zones were identified as follows:

- Risk characteristics:
  - Probability: likelihood of risk occurrence (C1)
  - Detectability: the organization’s ability to detect the risk (C2)
  - Manageability: the organization’s ability to manage the risk (C3)
- Risk impact zones:
  - Cost: financial impact of the risk(C4)
  - Time: impact on project schedule(C5)
  - Quality: impact on overall project quality(C6)

To complete the risk planning and identification phase, the interdependencies among risks and potential response strategies were identified through three iterations of the Delphi method, which facilitated the aggregation of expert opinions. The resulting list of risk interdependencies and response strategies is presented in Tables 6 and 9, respectively.

Another important consideration is the interdependency among risks. Like other types of information, risk interdependencies were identified during the planning and identification stage. The interdependencies identified for this project are presented in Table 6.

In the risk assessment stage, the identified risks are analyzed and evaluated against established criteria, including characteristics and impact zones. Before calculating the individual risk scores, the weights of these criteria must be determined and documented. This is done using the F-SWARA method. The results of the F-SWARA analysis are presented in Tables 10 and 11.

**Table 11:** F-SWARA, experts’ opinions on the criteria.

	C1 to C4	C5 to C1	C3 to C5	C2 to C3	C6 to C2
**E1**	(1,1,1)	(1,1,1)	(2/3,1,3/2)	(1,1,1)	(2/5,1/2,2/3)
**E2**	(2/3,1,3/2)	(1,1,1)	(2/5,1/2,2/3)	(2/3,1,3/2)	(2/5,1/2,2/3)
**E3**	(1,1,1)	(2/3,1,3/2)	(2/7,1/3, 2/5)	(2/7,1/3, 2/5)	(2/7,1/3, 2/5)

Source: Research findings.

As shown in Table 11, the criteria are ranked as follows: C4 > C1 > C5 > C3 > C2 > C6. This table also indicates the relative preferences among the subsequent criteria. Table 10 presents the final weights of the criteria, calculated according to the F-SWARA method.

**Fig 5:**
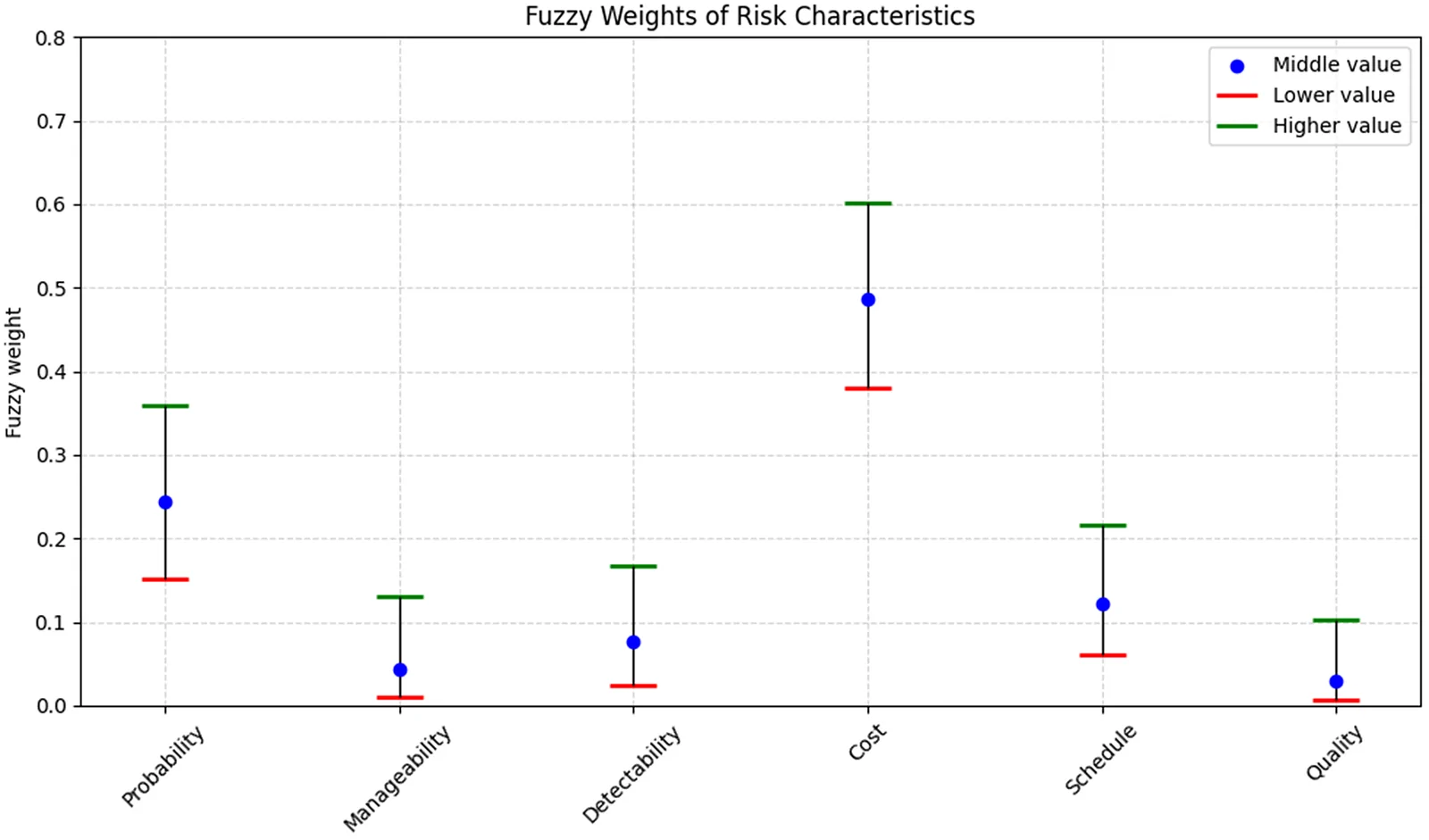
Fuzzy weights of risk criteria. Source: Research findings.

The F-SWARA results in Table 10 indicate that cost (C4) and probability (C1) are the most important criteria, which aligns with expectations from construction risk literature (e.g., [7,8,15,32,35]). The use of F-SWARA over other weighting methods (e.g., fuzzy AHP) was supported by a 30% reduction in expert elicitation time during the Delphi process. In contrast, detectability (C2) and quality (C6) are perceived as having the least impact on risk value, as clearly illustrated in Fig 5.

Next, the decision matrix is established using project documentation and expert estimates. The fuzzy decision matrix, which incorporates the values of the identified risks, is populated using the linguistic scale presented in Table 5. These values are assigned based on the nature of the criteria and the orientation of each risk. The actual estimates of the primary risk parameters —cost, delay, and quality—are also included for the modeling stage. The final decision matrix is provided in Table A1 in the S1 Appendix.

**Fig 6:**
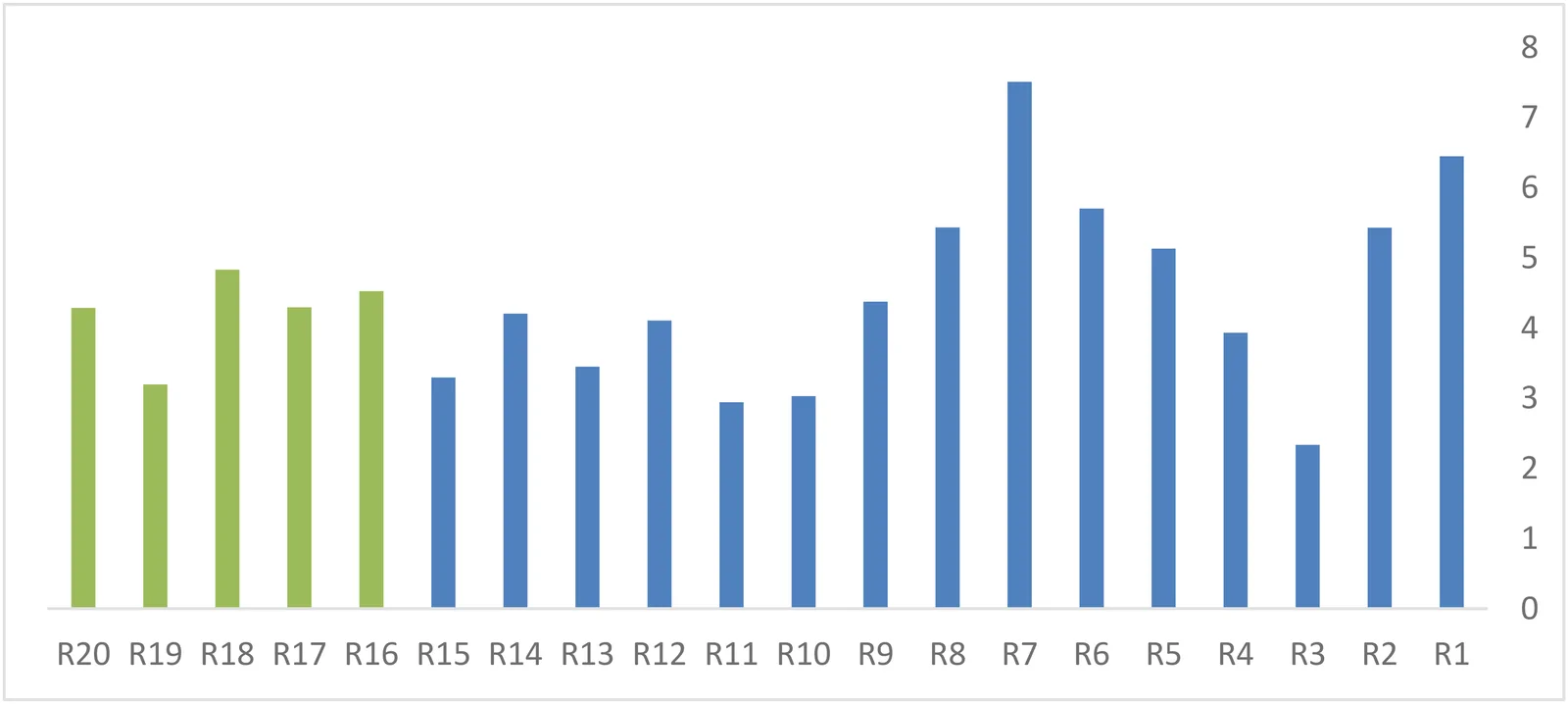
Risk values (weighted sum of risks) for each of the 20 identified risks. Values represent defuzzified risk scores (Eq. 5) ranging from 0 to 100. Blue bars indicate threats (negative risks), green bars indicate opportunities (positive risks). Source: Research findings.

In the final step of the second stage, the fuzzy decision matrix is used. Using the fuzzy criterion weights, the weighted sum for each risk is calculated with the Fuzzy Weighted Sum Method (F-WSM). The combined values for threats and opportunities are 67.346 and 21.138, respectively, yielding a total value of 46.208 units. Fig 6 illustrates the calculated values for each risk. The different risk groups show similar aggregate values, each around 20.00. Technical and technological risks scored the highest aggregate value (25.09), while management-related risks scored the lowest (18.86), though the values are relatively close.

**Fig 7:**
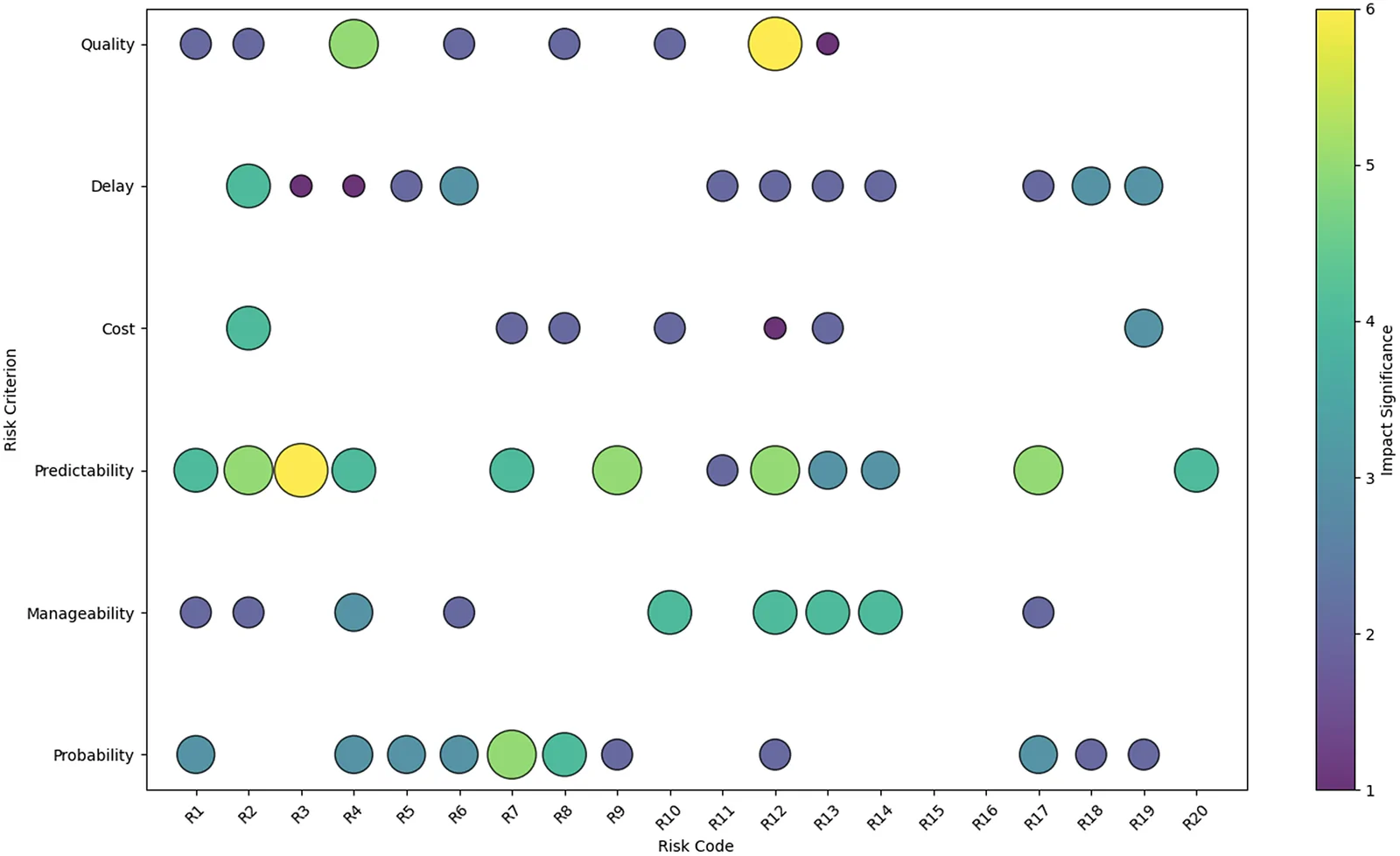
Effect of risk response strategies (S01–S12) on risk criteria (C1–C6). Cell colors indicate effect magnitude: Purple = low effect, Green: medium effect, Yellow = high effect. Linguistic terms (N, VL, L, BM, M, AM, H, VH, C) follow Table 5. Source: Research findings.

The ranking of risks presented in Fig 6 is derived from the systematic application of F-SWARA (for criteria weighting, Tables 10–11) and F-WSM (for risk scoring, Table A1 and Eqs. (6)– (11)). This quantitative ranking provides the empirical basis for the optimization model in Stage 3, which selects risk response strategies. With the risk scores and planning-stage information in hand, the third stage focuses on selecting an optimal set of risk response strategies for the project. As shown in Table 9, a list of candidate responses has been identified. However, implementing these responses incurs costs and requires time. The model involves a trade‑off between response requirements and risk mitigation, subject to constraints that risk values remain below the tolerance level and that response costs and time stay within the budget and available project float time. Detailed information on the risk responses—including their cost and time requirements, and their effects on various risks (using the linguistic terms in Table 5)—is provided in Tables A2 and A3 in the S1 Appendix. The potential effects of the response strategies on risks are also illustrated in Fig 7.

According to the model, if the criterion of a given risk exceeds a specified threshold, it may impact the criterion of another risk with which it shares a dependent relationship. In this case, the source risk influences the target risk: if the source risk’s criterion surpasses the threshold, the target risk’s criterion changes by a certain percentage (increase or decrease). The final consideration concerns overlap between risk response strategies. Some responses overlap in their effects, or other conditions prevent their simultaneous selection, adding further constraints to the model. The overlapping response strategies are:

- S01 and S02
- S08 and S09
- S04, S07 and S12

**Fig 8:**
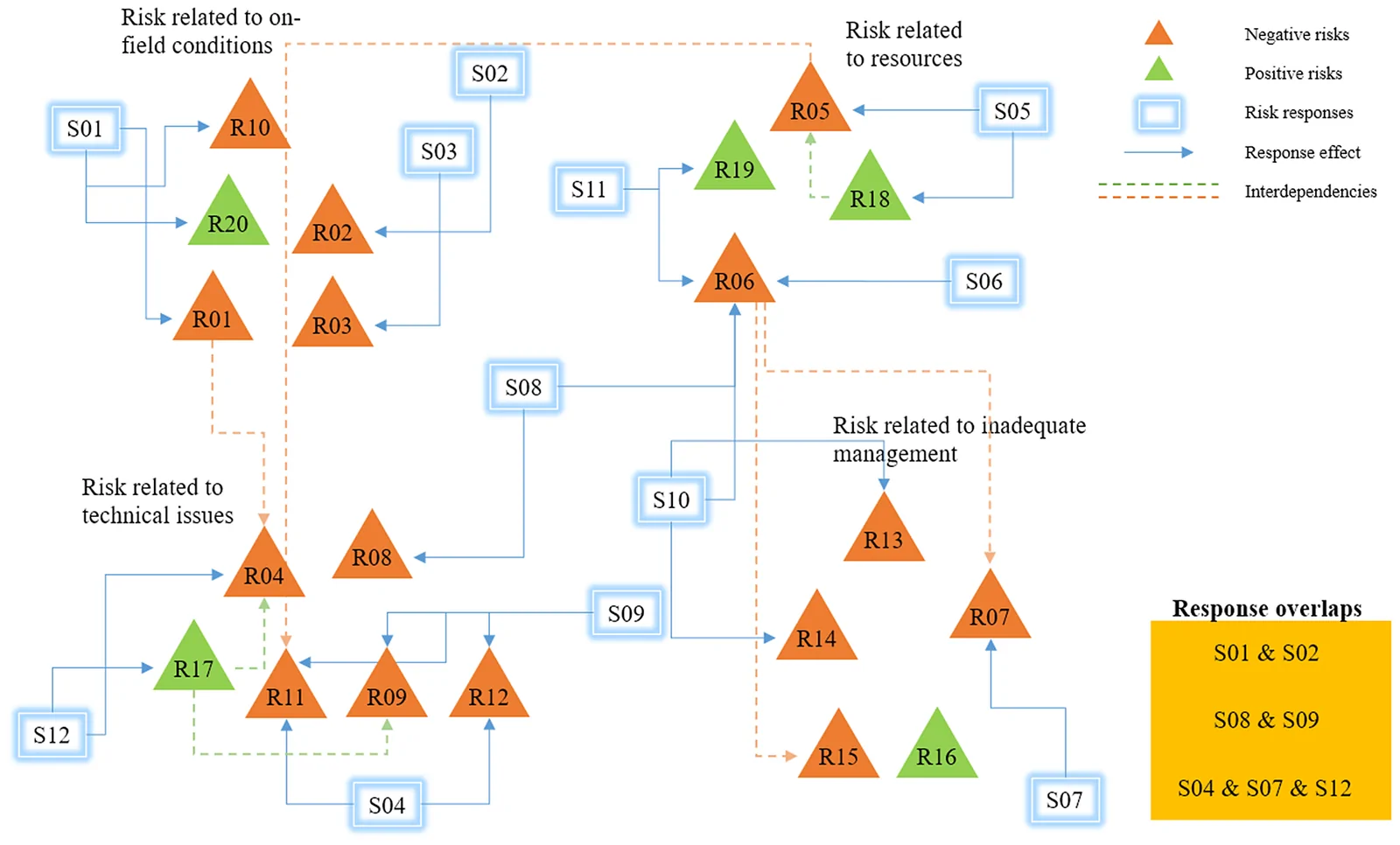
Network visualization of risks (triangles), response strategies (rectangles), directed arrows (response effects), interdependencies (dashed lines). Red triangles = threats, green triangles = opportunities, blue rectangles = selected responses. Source: Research findings.

Fig 8 provides a comprehensive visualization of the optimization problem, showing the relationships among the 20 identified risks (R01–R20), the 12 candidate response strategies (S01–S12), and the risk interdependencies (Table 6). Negative and positive risks are distinguished by color. Risks are grouped into four categories and placed at the four corners accordingly. Several response strategies affect multiple risks, and overlapping responses are also indicated.

As shown in Fig 8, several patterns emerge that are specific to the LPG pipeline construction context. First, technical and technological risks (R04, R08, R09, R11, R12, R17) exhibit the highest degree of interconnectedness, with multiple outgoing and incoming interdependency arrows. This finding aligns with prior pipeline construction studies [19,34,50,63], which identified technical failures (e.g., welding defects, corrosion) as primary risk drivers with cascading effects on schedule and quality. Second, response strategies S02 (environmental impact mitigation), S05 (supply chain management), and S12 (R&D investment) show the broadest coverage across risk categories, each addressing risks from multiple thematic groups. This suggests that integrated responses are more efficient than siloed approaches, a finding that contrasts with traditional zonal-based methods [54,55], which treat risk categories independently. Third, the overlap constraints between response strategies (S01–S02, S08–S09, S04–S07–S12) are visually confirmed in Fig 8. Notably, S12 (R&D investment) overlaps with both S04 (quality control) and S07 (cost estimation), indicating that technological solutions require coordination with operational and financial measures.

Compared with expectations, the prominence of management-related risks (R07, R13–R15) as targets rather than sources of interdependencies was unexpected. This suggests that while management risks are often triggered by external factors, they serve as amplifiers of other risks, consistent with Zhang [19] who noted that “soft” risks propagate through project networks.

**Fig 9:**
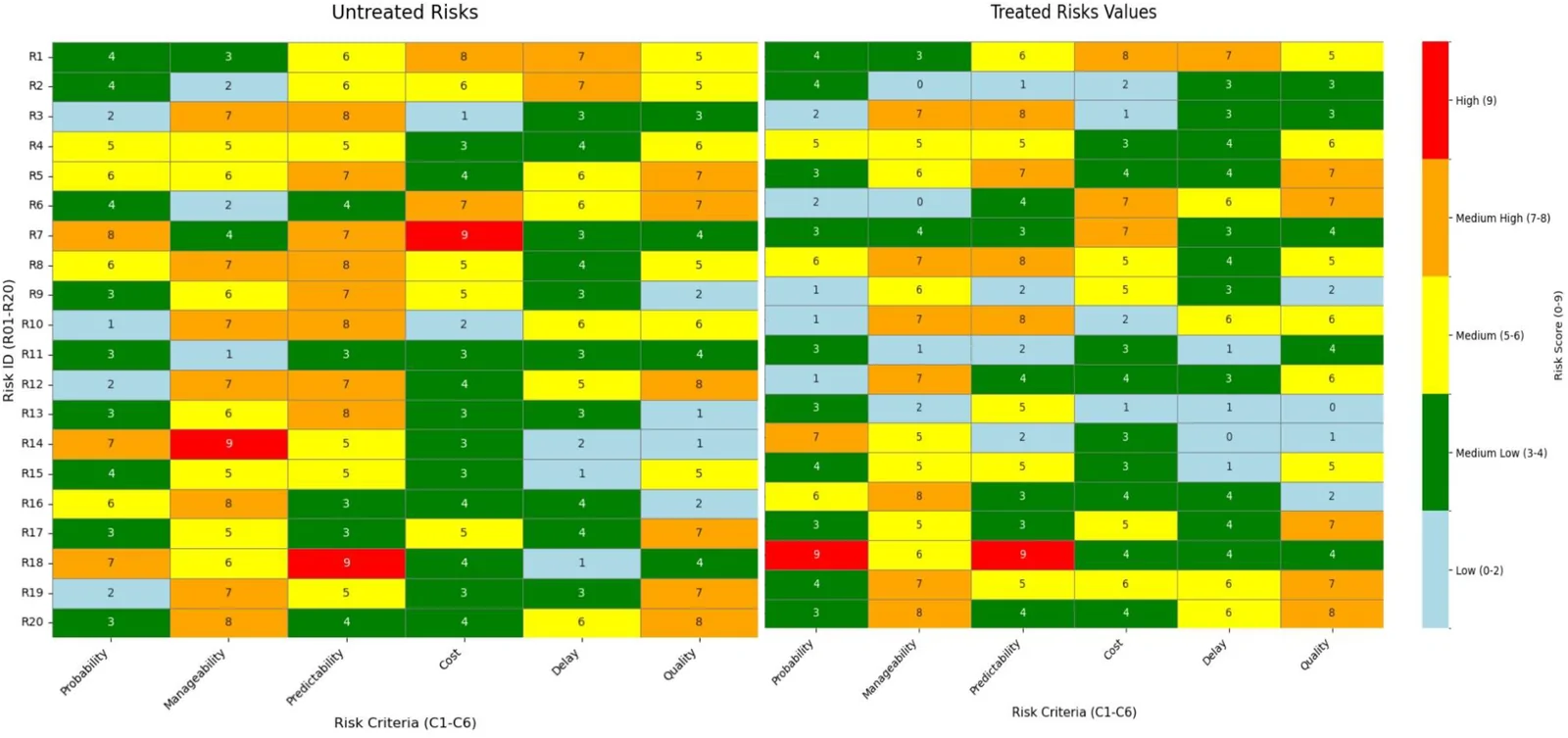
Changes in risk criteria values before and after response implementation. Heatmap displays crisp risk scores for 20 risks (rows) across 6 criteria (columns: C1–C6, defined as Probability, Detectability, Manageability, Cost impact, Time impact, and Quality impact, respectively). Risk Scores range from 1 (lowest) to 9 (highest risk contribution). Left panel: baseline scores (before responses). Right panel: residual scores after optimal responses. Color bar: blue = low risk, green = medium low risk, yellow = medium risk, orange = medium high risk, red = high risk. Source: Research findings.

With the data prepared and the model assumptions established, the optimization problem was solved using Python’s CPLEX library (Docplex model). The results indicate that the optimal strategy involves selecting 6 of the 12 available response strategies: S02, S05, S07, S09, S11, and S12. Implementing these responses reduces the total risk value from 46.208 to 28.243, yielding a 38.9% reduction in total risk value while satisfying all risk tolerances. The effects of the responses on the risk criteria are illustrated in Fig 9, which includes two heatmaps showing the values of the crisp decision matrix before and after response implementation.

In Fig 9, the horizontal axis presents the risk characteristics, while the vertical axis depicts the 20 identified risks. Each cell contains a numerical value on a scale from 1 to 9, corresponding to the significance of the respective risk characteristic. The left heatmap represents the initial (baseline) risk state, whereas the right heatmap illustrates changes after implementing the selected response strategies. The numerical values in Fig 9 are defuzzified risk scores (range 1–9) derived from the fuzzy decision matrix (Table A1) using Eq. (5). Lower values indicate lower risk contribution for threats or higher opportunity potential for positive risks.

A comparison between the left and right panels reveals that the selected response strategies reduced risk scores across all criteria. The most substantial reductions were observed in C4 (cost impact) and C1 (probability), consistent with the criteria weights shown in Table 10. For instance, R07 (cost estimation errors) decreased from 8.5 to 3.2, representing a 62% reduction, primarily attributable to the selection of S07 (enhanced cost estimation). By contrast, detectability (C2) showed the smallest absolute reduction, reflecting its lower weight in the F-SWARA analysis. This study presents an integrated approach to project risk management, encompassing three stages: planning and identification, assessment, and selection of a risk response strategy aimed at optimizing the total risk value of the project. An empirical case involving the construction of an LPG pipeline is employed to test the model. Initially, a set of relevant risks was identified from the literature. Subsequently, using a combination of checklists and three iterations of the Delphi method, the required information was collected, resulting in the identification of 20 risks and 12 response strategies. Next, the risk characteristics were weighted using the fuzzy SWARA (F-SWARA) method. Among the determined characteristics, cost and probability held the highest weight, while the impact on quality received the lowest. Thereafter, using the acquired risk weights, the fuzzy weighted sum model (F-WSM) was employed to evaluate each risk and assign a score reflecting its relative significance. Among the identified risks, R01 and R07 were found to be the most threatening, whereas R03 had the smallest impact. The positive risks were determined to have relatively equal values. Finally, the value of each risk was inserted into the mixed-integer linear programming (MILP) model, to optimize the overall risk value using the available risk response strategies.

**Fig 10:**
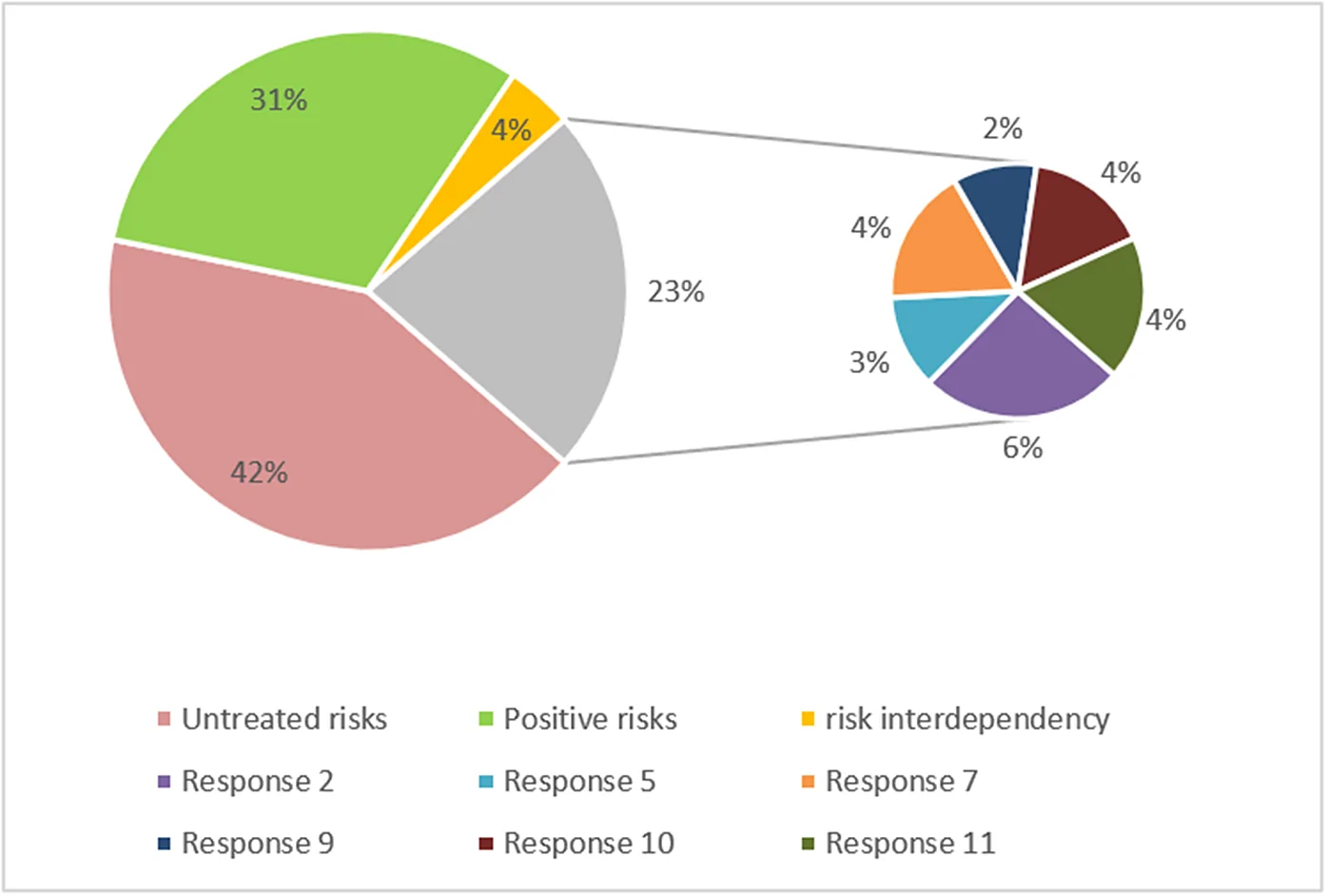
Breakdown of total risk value reduction, from 46.208 to 28.243 units (a 38.9% decrease). The stacked bars illustrate the contributions of positive risks (teal), selected response strategies (grey), and risk interdependencies (orange). Source: Research findings.

Of the 12 available response strategies, 6 were selected, yielding a 38.9% reduction in total risk value. In addition to the selected response strategies, risk interdependencies and positive risks also contributed to the observed change in total risk value. A detailed breakdown of the risk value is provided in Fig 10.

Fig 10 presents the risk value breakdown following the optimization and risk response selection. The total risk value decreased from 46.208 to 28.243 units, representing a 38.9% reduction. The decomposition reveals that positive risks (opportunities, R16–R20) contributed a reduction of 21.138 units, making them the largest single mitigating factor. Risk response strategies contributed 12.345 units (23% of the total reduction), while risk interdependencies had a net reduction of only 0.482 units (4%) due to conflicting effects—some interdependencies increased risk while others decreased it. This indicates that investing in opportunity exploitation is as valuable as risk mitigation.

The mitigation contribution of the six selected responses is further detailed in the smaller pie chart on the right. The impact of individual responses was relatively consistent, with S02 having the greatest impact and S09 the least. Among the selected responses, two (S10 and S11) aimed at exploiting opportunities, while the remainder focused on threat avoidance or mitigation. Risk responses were unevenly distributed across risk groups: resource-related and technical risks were addressed far more extensively than management-related and on-site risks, likely due to the nature of the responses and their individual value optimization.

Notably, the impact of positive risks exceeded expectations, given prior pipeline studies [34,50] that typically emphasize threats over opportunities. This finding suggests that LPG pipeline projects may have underutilized potential for opportunity exploitation, particularly in areas such as bulk material purchasing (R18) and technological advancements (R17). Practitioners should therefore allocate resources not only to threat mitigation but also to capitalizing on opportunities.

### Sensitivity analysis

To assess the robustness of the proposed model, a sensitivity analysis was conducted by systematically varying key input parameters. Two sensitivity analyses were conducted to validate model robustness.

**Fig 11:**
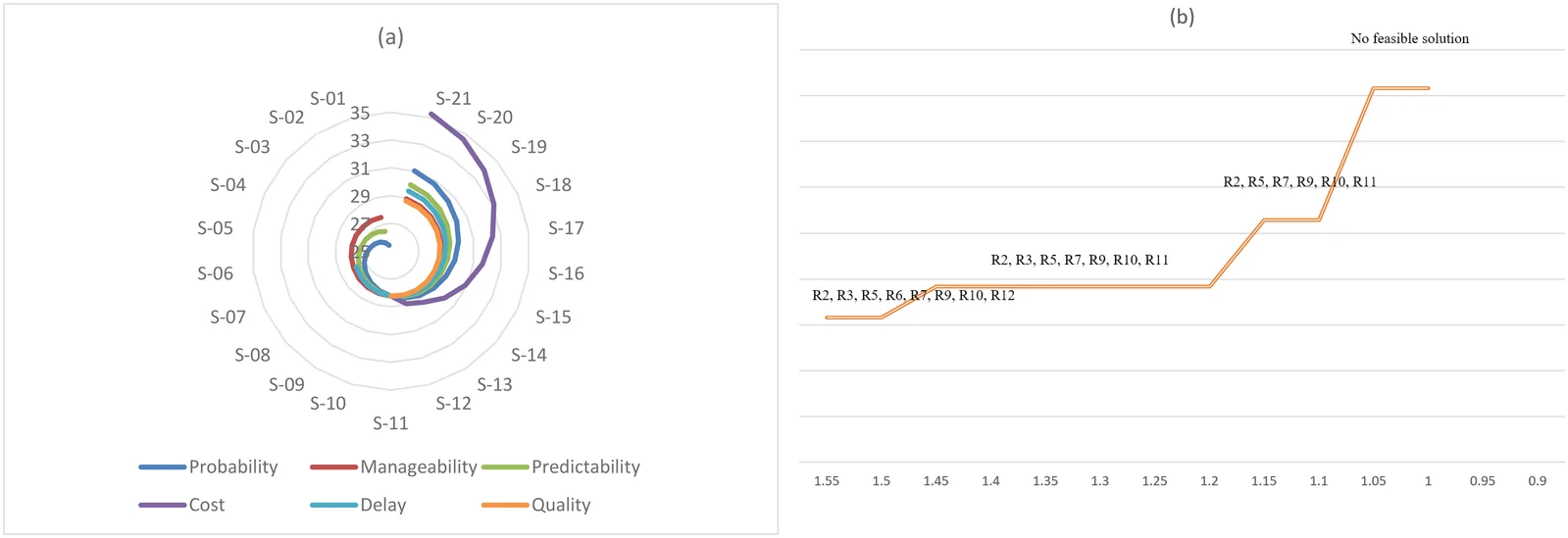
Sensitivity analysis results: (a) effect of varying risk criteria values (±50%) on total risk value; (b) effect of varying response requirements (±50%) on number of selected responses. Dashed lines indicate baselines. Shaded regions show stable ranges. Source: Research findings.

First, the values of the risk criteria were adjusted across a range of [−50%, + 50%] over 21 scenarios. After excluding infeasible scenarios, the total risk value was assessed, and the results are illustrated in Fig 11(a). The total risk value remained within ±15% of baseline for variations up to ±30%, with infeasibility occurring only at extreme deviations (beyond +40% or below −40%). This suggests that the optimal solution is robust under realistic project uncertainty. Cost (monetary impact) proved to be the most sensitive parameter, followed by probability. These two parameters most significantly affect the outcome of the programming model, consistent with the recognition that cost and probability are fundamental components of risk scores.

Second, to assess the sensitivity of the selected risk responses, the monetary and time requirements of the response strategies were varied across a [−50%, + 50%] range in 11 scenarios. The results of this sensitivity analysis are presented in Fig 11(b).

The selected response set (S02, S05, S07, S09, S11, S12) remained stable for requirement reductions up to 40% and increases up to 20%. Beyond these thresholds, additional responses became feasible when requirements were reduced by 40% or more, while increasing requirements by more than 30% resulted in no feasible solution. Under baseline conditions, the model selects the original set of risk response strategies. When constraints are tightened, no feasible solution exists. Conversely, when constraints are relaxed (i.e., additional time and budget are allocated), the model automatically selects the next best responses—in this case, S03 and S11. Notably, overlapping responses did not replace the originally selected strategies, confirming that the chosen responses (S02, S05, S07, S09, S11, S12) are robustly optimal under moderate parameter variations. These results confirm that the model is robust to moderate input variations and that the selected response set is near-optimal.

### Model validation

To validate the proposed integrated optimization framework, three complementary approaches were employed.

**Face validity with experts.** The optimal set of six selected response strategies (S02, S05, S07, S09, S11, S12) was presented to the three domain experts who participated in the Delphi process. All three experts independently confirmed that the selected strategies align with practical expectations for an LPG pipeline construction project. Specifically, they noted that S02 (environmental impact mitigation) and S09 (preventive maintenance) are standard practices, while S11 (local partnership programs) and S12 (R&D investment) represent innovative yet appropriate strategies for opportunity exploitation.

**Comparison with a rule-based benchmark.** To assess whether the optimization model provides superior selection compared to a simpler heuristic, a rule-based benchmark was implemented: selecting the six response strategies that address the largest number of high-risk criteria. This benchmark yielded only a 24.1% reduction in total risk value, compared to 38.9% achieved by the optimization model. This confirms the superiority of the optimization-based selection over a naive heuristic.

**Stability against alternative weighting schemes.** To evaluate whether the risk ranking is sensitive to the choice of weighting method, the F-SWARA weights were compared with equal weights and with weights derived from Fuzzy AHP (using a subset of criteria to reduce expert burden). The Spearman rank correlation between F-SWARA and Fuzzy AHP rankings was 0.89 (p < 0.01), and 0.82 with equal weights, indicating strong stability. Thus, the risk prioritization is robust to alternative weighting schemes.

Collectively, these validation approaches demonstrate that the proposed model produces credible, robust, and practically relevant results.

## Discussion

The finding that technical risks exhibit the highest degree of interdependency (Fig 8) aligns with Zhang [19] and Safaeian et al. [63], who demonstrated that technical failures in construction projects create cascading effects. However, our results diverge from previous studies in two respects. First, the substantial contribution of positive risks (21.1 units, or 46% of total risk reduction) exceeds the typical treatment of opportunities in standard risk management frameworks such as the PMBOK® Guide [3], which emphasizes threat mitigation. Second, the overlap constraint between S12 (R&D investment) and S04 (quality control) suggests that technological and operational responses are not independent—a relationship not captured in conventional optimization models [37,61].

For practitioners, the results indicate that: (a) technical risks (welding, corrosion, equipment failure) should be prioritized for interdependency management; (b) response strategies should be selected in bundles rather than individually, given the overlap constraints identified; and (c) opportunity exploitation (e.g., bulk purchasing, technological advancements) offers untapped risk reduction potential. The selected six responses (S02, S05, S07, S09, S11, S12) provide a template for similar pipeline projects, though customization is required for local conditions.

The integrated framework successfully linked risk assessment outputs to response selection, addressing a key gap identified in the literature (Section 2). The use of F-SWARA for criteria weighting proved appropriate given the small expert panel, but future applications should consider larger panels for enhanced reliability. The MILP formulation guaranteed global optimality, though computational time scaled exponentially with problem size; for projects with more than 50risks, heuristic approaches may be necessary.

This study has several limitations. The single-case design limits generalizability; cross-case comparisons across multiple pipeline projects are needed. The expert panel (n = 3), while justified, introduces potential bias; future studies should employ larger panels and inter-rater reliability metrics beyond Spearman’s ρ. Finally, the model assumes deterministic risk interdependency thresholds; a stochastic extension would better capture real-world uncertainty.

This study contributes to the existing body of literature by introducing an integrated systematic optimization approach to project risk management, a critical domain within project management scholarship. The proposed framework synthesizes risk planning and identification, risk analysis and evaluation, and the selection of risk response strategies—an area that has not been adequately addressed in prior research. Notably, the planning phase incorporates essential considerations often overlooked in the literature, such as the duality of risk effects (both negative and positive) and the interdependencies among risks.

The analysis and evaluation phases examined the characteristics of the identified risks and the relevant project parameters they influence, referred to as impact zones. Multi-Criteria Decision-Making (MCDM) methods were employed to weigh and assess these risks effectively. The selection of risk response strategies was achieved through the optimization of risk values within project constraints, distinguishing this study from previous works that typically treat risk assessment and response selection as separate processes.

Additionally, this research addresses significant gaps in the literature, including the differentiation between threats and opportunities, the consideration of risk characteristics during risk evaluation, and the acknowledgment of risk interdependencies. To validate the proposed model, empirical data from an LPG pipeline construction project were collected and analyzed. The research question is addressed through the introduction of the framework and its empirical testing, thereby providing a comprehensive means to integrate both risk assessment and risk response selection.

## Conclusion

This study developed an integrated, optimization-oriented framework that links project risk assessment directly to the selection of risk response strategies. Rather than treating risk evaluation and response planning as separate activities, the proposed approach connects fuzzy multi-criteria risk assessment outputs to a mixed-integer linear programming (MILP) model. This enables the systematic and optimal selection of response actions under real-world constraints. The framework extends conventional risk assessment practices by incorporating multiple risk characteristics beyond probability and impact, and by explicitly accounting for risk interdependencies. Most importantly, it operationalizes risk management as a decision-support problem, where assessed risks are transformed into quantitative inputs for an optimization model. The framework comprises three stages: (1) risk planning and identification—gathering risks, responses, and relationships from literature, experts, and project documentation; (2) risk assessment—using F-SWARA for weighting and F-WSM for evaluation, incorporating characteristics beyond probability-severity (e.g., detectability, manageability); and (3) response selection—using a MILP model to choose optimal strategies under budget, time, tolerance, and interdependency constraints.

This framework advances beyond standard Fuzzy SWARA applications in three specific ways. First, it integrates Fuzzy SWARA with Fuzzy WSM and MILP into an end-to-end workflow, whereas most existing applications use Fuzzy SWARA only for criteria weighting. Second, it incorporates risk interdependencies (Table 6) and dual risk effects (threats and opportunities), which are absent from conventional Fuzzy SWARA implementations. Third, it explicitly links risk assessment outputs to response selection constraints (budget, time, tolerances), as shown in Eqs. (6)– (16). These integrations are novel within the project risk management domain, though the individual methods themselves are not new. The terminology “framework” is justified by this integration of multiple methods (F-SWARA, F-WSM, MILP) and constructs (risk characteristics, impact zones, interdependencies) into a coherent, replicable decision-support structure, further reinforced by empirical validation.

We reiterate that optimization is confined to the MILP‑based response selection stage (Subsections 3.5&3.6); the preceding risk assessment stages rely on MCDM methods (F‑SWARA and F‑WSM), not optimization. From an optimization perspective, the problem formulated in Subsections 3.5&3.6 is a combinatorial optimization problem with ${\text{2}}^{|S|}$possible subsets of risk response strategies, where |S|=12 in the presented case study (${\text{2}}^{\text{1}\text{2}}=\text{4}\text{0}\text{9}\text{6}$ possible combinations). The mixed‑integer linear programming (MILP) model efficiently searches this solution space and identifies the globally optimal subset without enumerating all possibilities, solved to optimality using CPLEX.

Applied to a real-world LPG pipeline project, the framework yielded several domain-specific findings. First, 20 risks were identified, with technical risks (R04, R08, R09, R11, R12, R17) showing the highest interconnectedness—confirming welding defects, corrosion, and equipment failure as primary drivers, consistent with prior studies [34,50]. The most critical risks were R07, R06, and R01, two of which were addressed by the selected responses, which targeted resource-related factors (raw material delays, labor shortages) and technical issues (accidents, equipment failures), reflecting the resource-intensive and operationally complex nature of LPG projects. Second, F-SWARA weighting indicated that cost impact (C4: 0.381–0.601) and probability (C1: 0.152–0.360) dominate risk evaluation, while detectability (C2: 0.010–0.131) and quality impact (C6: 0.006–0.102) were deemed less significant—consistent with practitioners’ focus on budget overruns over quality deviations. Third, the optimization model selected six responses (S02, S05, S07, S09, S11, S12), achieving a 38.9% reduction in total risk value (from 46.208 to 28.243 units). The optimal solution includes a balanced mix of response types, consisting of two exploitative, two mitigative, and two avoidance strategies. This demonstrates that effective risk management requires a diversified portfolio beyond traditional mitigation. Notably, positive risks contributed 21.138 units of this reduction, exceeding the 12.345 units from selected responses, suggesting underutilized opportunity exploitation.

Fourth, sensitivity analyses confirmed stability for ±30% variations in risk criteria and between −40% and +20% in response requirements, indicating robustness to moderate uncertainty. Validation through expert face validation, a rule-based benchmark (24.1% risk reduction vs. 38.9% from optimization), and alternative weighting schemes (Spearman rank correlation more than 0.82) further supported the model’s reliability.

Finally, the case study underscores the importance of aligning responses with risk attributes: resource-related risks were best managed through exploitative and mitigative strategies, while technical risks required avoidance or targeted mitigation. These findings offer practical guidance for project managers by demonstrating how risk characteristics can systematically inform response selection.

### Practical and theoretical implications

This study contributes to the project risk management literature in four principal ways. First, it integrates risk assessment and response selection into a unified MILP model with fuzzy MCDM (F-SWARA and F-WSM), enabling more accurate and robust findings than sequential approaches. Second, it incorporates three overlooked constructs—dual risk effects, characteristics beyond probability-severity (detectability, manageability, predictability), and interdependencies—within a quantitative optimization framework. Third, empirical validation on LPG pipeline data shows that positive risks contributed most to the 38.9% risk reduction, challenging threat-centric perspectives and highlighting opportunity exploitation. Fourth, it demonstrates effective F-SWARA/F-WSM synergy while maintaining linearity to avoid computational complexity—a balance not systematically addressed previously.

Beyond theory, the framework offers practical value as a decision-support tool. Key insights for practitioners include: transforming expert judgments into feasible strategies (six of twelve responses selected, 38.9% reduction); targeted resource allocation via risk characteristics and impact zones; evidence-based prioritization (cost and probability most weighted); accounting for interdependencies and prioritizing technical risks (welding, corrosion, equipment failure); bundling responses to avoid overlaps (constraints: S01–S02, S08–S09, S04–S07–S12); exploiting opportunities (R16–R20), which contributed 46% of reduction; sensitivity analysis for robustness; computational efficiency; adaptability to other infrastructure and non-construction domains; integration with PMIS/dashboards; and a customizable template for similar projects.

### Research limitations and future research

The proposed framework is presented as a case study application of integrated risk assessment and optimization methods, not as a new algorithmic contribution to optimization theory. Consequently, several limitations must be acknowledged.

The LPG pipeline project examined is relatively generic, and the framework was tested on only one case. The risk patterns, weights, and selected responses may not generalize to other projects with different geographical, regulatory, or organizational contexts. Consequently, findings and applicability may vary when applied to more complex projects with diverse risk profiles—a notable limitation.

A key limitation is the use of only three experts for risk identification, weighting, and evaluation. While justified by the specialized nature of LPG pipeline construction and the seniority of the experts (15–22 years of experience), organizational constraints prevented input from operational staff, allowing only three main project overseers to participate. This small sample limits the statistical generalizability of the risk scores, selected responses, and results more broadly. The results should therefore be interpreted as illustrative rather than statistically generalizable, and this study should be viewed as a narrow proof-of-concept demonstration. Although inter-rater reliability checks (Spearman’s ρ = 0.82–0.91) indicated acceptable agreement, and the Delphi method with fuzzy logic helped mitigate individual biases, larger panels would be preferable in future work. Future studies should evaluate the framework using larger, more heterogeneous expert groups to assess robustness across organizational and project contexts, and should employ inter-rater reliability analysis to strengthen the validity of the results.

The study did not benchmark the proposed MILP approach against other optimization methods (e.g., genetic algorithms, simulated annealing) or alternative MCDM techniques (e.g., fuzzy AHP, TOPSIS, VIKOR). Such comparisons would strengthen claims of superiority or suitability.

Another limitation is methodological: the use of the Weighted Sum Model (WSM) for risk evaluation. Compared to non-linear MCDM methods such as fuzzy TOPSIS or WASPAS, WSM is simpler and assumes criteria independence. However, WSM was deliberately chosen to preserve linearity in the subsequent MILP optimization. If future research adopts non-linear MCDM (e.g., fuzzy TOPSIS) or probabilistic methods (e.g., Bayesian networks), response selection would require metaheuristic algorithms (e.g., NSGA-II, particle swarm optimization) instead of exact MILP solvers, introducing a trade-off between optimality guarantee and modeling flexibility. Additionally, the model assumes fixed risk interdependency thresholds and linear relationships; a stochastic extension would better capture real-world uncertainty.

Based on the limitations of this study, four future research directions are proposed. First, the framework should be applied prospectively to multiple pipeline projects to validate the 38.9% risk reduction claim and assess generalizability across varying contexts. Second, comparative studies with alternative optimization and MCDM methods (e.g., fuzzy TOPSIS, WASPAS, NSGA-II, particle swarm optimization) should be conducted to benchmark the MILP approach. Third, the linear programming model could be extended to a stochastic formulation to handle uncertainty in interdependency thresholds and other inputs, enhancing robustness. Fourth, the expert panel should be expanded to include broader stakeholders—contractors, regulators, and community representatives—to capture diverse perspectives and improve validity. Ultimately, the optimization model could evolve to incorporate more nuanced real-world considerations, such as interdependencies among response strategies.

## Supporting information

S1 File Supplementary Code. This file provides the complete Python code used to implement the proposed integrated framework. (RAR)

S2 File Supplementary Data. This file provides the full dataset used in the analyses needed to reproduce all reported results. (RAR)

S1 Appendix Supplementary data. This appendix provides the full dataset used in the analyses needed to reproduce all reported results. (DOCX)
