# Supplementary material for: An integrated fuzzy multi-criteria decision making and mixed-integer linear programming optimization framework for project risk assessment and response strategy selection: A case study of a liquefied petroleum gas pipeline construction project
Source: PLoS One. 2026 Sep 24;21(9):e0357560. doi: 10.1371/journal.pone.0357560 (PMC13606970; doi:10.1371/journal.pone.0357560)
Supplement: Supplementary file 1 [file pone.0357560.s003.docx]

**Table A.1. Detailed information on the risk fuzzy decision matrix**

|  |  |  | **Risk Characteristics** | | | | |  |  |  |  | **Risk impact** | | | | |  |  | **Estimated crisp data** | | |
| --- | --- | --- | --- | --- | --- | --- | --- | --- | --- | --- | --- | --- | --- | --- | --- | --- | --- | --- | --- | --- | --- |
|  | Probability | | | **Manageability** | | | **Predictability** | | | **Cost** | | | **Delay** | | | **Quality** | | | **Cost** | **Delay** | **Quality (%)** |
| **R1** | 3 | 4 | 5 | 2 | 3 | 4 | 5 | 6 | 7 | 7 | 8 | 9 | 6 | 7 | 8 | 4 | 5 | 6 | 8 | 1.5 | 5% |
| **R2** | 3 | 4 | 5 | 1 | 2 | 3 | 5 | 6 | 7 | 5 | 6 | 7 | 6 | 7 | 8 | 4 | 5 | 6 | 5.5 | 1.5 | 6% |
| **R3** | 1 | 2 | 3 | 6 | 7 | 8 | 7 | 8 | 9 | 1 | 1 | 2 | 2 | 3 | 4 | 2 | 3 | 4 | 1 | 0.5 | 3% |
| **R4** | 4 | 5 | 6 | 4 | 5 | 6 | 4 | 5 | 6 | 2 | 3 | 4 | 3 | 4 | 5 | 5 | 6 | 7 | 3 | 1 | 6% |
| **R5** | 5 | 6 | 7 | 5 | 6 | 7 | 6 | 7 | 8 | 3 | 4 | 5 | 5 | 6 | 7 | 6 | 7 | 8 | 4.5 | 1.5 | 7% |
| **R6** | 3 | 4 | 5 | 1 | 2 | 3 | 3 | 4 | 5 | 6 | 7 | 8 | 5 | 6 | 7 | 6 | 7 | 8 | 6.5 | 1.5 | 8% |
| **R7** | 7 | 8 | 9 | 3 | 4 | 5 | 6 | 7 | 8 | 8 | 9 | 9 | 2 | 3 | 4 | 3 | 4 | 5 | 8.5 | 0.1 | 4% |
| **R8** | 5 | 6 | 7 | 6 | 7 | 8 | 7 | 8 | 9 | 4 | 5 | 6 | 3 | 4 | 5 | 4 | 5 | 6 | 5 | 0.5 | 6% |
| **R9** | 2 | 3 | 4 | 5 | 6 | 7 | 6 | 7 | 8 | 4 | 5 | 6 | 2 | 3 | 4 | 1 | 2 | 3 | 4.5 | 0.5 | 2% |
| **R10** | 1 | 1 | 2 | 6 | 7 | 8 | 7 | 8 | 9 | 1 | 2 | 3 | 5 | 6 | 7 | 5 | 6 | 7 | 2 | 1.5 | 7% |
| **R11** | 2 | 3 | 4 | 1 | 1 | 2 | 2 | 3 | 4 | 2 | 3 | 4 | 2 | 3 | 4 | 3 | 4 | 5 | 3 | 0.5 | 4% |
| **R12** | 1 | 2 | 3 | 6 | 7 | 8 | 6 | 7 | 8 | 3 | 4 | 5 | 4 | 5 | 6 | 7 | 8 | 9 | 3.5 | 1.5 | 9% |
| **R13** | 2 | 3 | 4 | 5 | 6 | 7 | 7 | 8 | 9 | 2 | 3 | 4 | 2 | 3 | 4 | 1 | 1 | 2 | 3 | 1 | 1% |
| **R14** | 6 | 7 | 8 | 8 | 9 | 9 | 4 | 5 | 6 | 2 | 3 | 4 | 1 | 2 | 3 | 1 | 1 | 2 | 3 | 0.5 | 1% |
| **R15** | 3 | 4 | 5 | 4 | 5 | 6 | 4 | 5 | 6 | 2 | 3 | 4 | 1 | 1 | 2 | 4 | 5 | 6 | 3 | 0.1 | 6% |
| **R16** | 5 | 6 | 7 | 7 | 8 | 9 | 2 | 3 | 4 | 3 | 4 | 5 | 3 | 4 | 5 | 1 | 2 | 3 | 4 | 1 | 2% |
| **R17** | 2 | 3 | 4 | 4 | 5 | 6 | 2 | 3 | 4 | 4 | 5 | 6 | 3 | 4 | 5 | 6 | 7 | 8 | 5.5 | 1 | 8% |
| **R18** | 6 | 7 | 8 | 5 | 6 | 7 | 8 | 9 | 9 | 3 | 4 | 5 | 1 | 1 | 2 | 3 | 4 | 5 | 4 | 0.1 | 4% |
| **R19** | 1 | 2 | 3 | 6 | 7 | 8 | 4 | 5 | 6 | 2 | 3 | 4 | 2 | 3 | 4 | 6 | 7 | 8 | 3 | 0.5 | 8% |
| **R20** | 2 | 3 | 4 | 7 | 8 | 9 | 3 | 4 | 5 | 3 | 4 | 5 | 5 | 6 | 7 | 7 | 8 | 9 | 4 | 1.5 | 9% |
|  |  |  |  |  |  |  |  |  |  |  |  |  |  |  | Risk tolerance 🡪 | | | | 30M | 6 months | 40% |

Source: Research findings

**Table A.2. Impact of response strategies on risks**

|  | **Risk Characteristics** | | | **Risk impact** | | |
| --- | --- | --- | --- | --- | --- | --- |
|  | **Probability** | **Manageability** | **Predictability** | **Cost** | **Delay** | **Quality** |
| **R1** | (L)S1 | (VL)S1 | (BM)S1 |  |  | (VL)S1 |
| **R2** |  | (VL)S2 | (M)S2 | (BM)S2 | (BM)S2 | (VL)S2 |
| **R3** |  |  | (AM)S3 |  | (N)S3 |  |
| **R4** | (L)S12 | (L)S12 | (BM)S12 |  | 1S12 | (M)S12 |
| **R5** | (L)S5 |  |  |  | (VL)S5 |  |
| **R6** | (N)S8 + (VL)S11 | (VL)S10 |  |  | (L)S6 | (VL)S6 |
| **R7** | (M)S7 |  | (BM)S7 | (VL)S7 |  |  |
| **R8** | (BM)S8 |  |  | (VL)S8 |  | (VL)S8 |
| **R9** | (VL)S9 |  | (M)S9 |  |  |  |
| **R10** |  | (BM)S1 |  | (VL)S1 |  | (VL)S1 |
| **R11** |  |  | (N)S4+(N) S9 |  | (VL)S9 |  |
| **R12** | (N)S4+(N)S9 | (BM)S4 | (VL)S4+(L) S9 | (N)S4 | (VL)S9 | (BM)S4+(VL)S9 |
| **R13** |  | (BM)S10 | (L)S10 | (VL)S10 | (VL)S10 | S10 |
| **R14** |  | (BM)S10 | (L)S10 |  | (VL)S10 |  |
| **R15** |  |  |  |  |  |  |
| **R16** |  |  |  |  |  |  |
| **R17** | (L)S12 | (VL)S12 | (M)S12 |  | (VL)S12 |  |
| **R18** | (VL)S5 |  |  |  | (L)S5 |  |
| **R19** | (VL)S11 |  |  | (L)S11 | (L)S11 |  |
| **R20** |  |  | (BM)S1 |  |  |  |

Source: Research findings

**Table A.3. Requirements of risk response strategies**

|  | **Cost of response strategy** | **Implementation time** |
| --- | --- | --- |
| **S1** | 3.8 | 2 |
| **S2** | 3.2 | 1 |
| **S3** | 0.5 | 0.5 |
| **S4** | 2.5 | 0.5 |
| **S5** | 0.6 | 0.5 |
| **S6** | 2 | 2 |
| **S7** | 0.7 | 1 |
| **S8** | 2.1 | 1.5 |
| **S9** | 1.1 | 1 |
| **S10** | 1 | 1 |
| **S11** | 0.3 | 0.5 |
| **S12** | 1 | 1 |
| Budget and float time available for response allocation Source: Research findings | 8M | 5 months |
